# The effect of residual palladium on the performance of organic electrochemical transistors

**DOI:** 10.1038/s41467-022-35573-y

**Published:** 2022-12-27

**Authors:** Sophie Griggs, Adam Marks, Dilara Meli, Gonzague Rebetez, Olivier Bardagot, Bryan D. Paulsen, Hu Chen, Karrie Weaver, Mohamad I. Nugraha, Emily A. Schafer, Joshua Tropp, Catherine M. Aitchison, Thomas D. Anthopoulos, Natalie Banerji, Jonathan Rivnay, Iain McCulloch

**Affiliations:** 1 Department of Chemistry, Chemistry Research Laboratory, University of Oxford, Oxford, OX1 3TA UK; 2 Department of Materials Science and Engineering, Northwestern University, Evanston, IL 60208 USA; 3 Department of Chemistry, Biochemistry and Pharmaceutical Sciences (DCBP), University of Bern, Freiestrasse 3, 3012 Bern, Switzerland; 4 Department of Biomedical Engineering, Northwestern University, Evanston, IL 60208 USA; 5 KAUST Solar Center (KSC), King Abdullah University of Science and Technology (KAUST), Thuwal, 23955-6900 Saudi Arabia; 6 Great Bay University, 523808 Dongguan, China; 7 Department of Earth System Science, Stanford University, Stanford, CA 94305 USA; 8 Physical Sciences and Engineering Division (PSE), KAUST Solar Center (KSC), King Abdullah University of Science and Technology (KAUST), Thuwal, 23955-6900 Saudi Arabia; 9 Research Center for Advanced Materials, National Research and Innovation Agency (BRIN), South Tangerang, Banten 15314 Indonesia; 10 Simpson Querrey Institute, Northwestern University, Chicago, IL 60611 USA

**Keywords:** Materials for devices, Conjugated polymers

## Abstract

Organic electrochemical transistors are a promising technology for bioelectronic devices, with applications in neuromorphic computing and healthcare. The active component enabling an organic electrochemical transistor is the organic mixed ionic-electronic conductor whose optimization is critical for realizing high-performing devices. In this study, the influence of purity and molecular weight is examined for a p-type polythiophene and an n-type naphthalene diimide-based polymer in improving the performance and safety of organic electrochemical transistors. Our preparative GPC purification reduced the Pd content in the polymers and improved their organic electrochemical transistor mobility by ~60% and 80% for the p- and n-type materials, respectively. These findings demonstrate the paramount importance of removing residual Pd, which was concluded to be more critical than optimization of a polymer’s molecular weight, to improve organic electrochemical transistor performance and that there is readily available improvement in performance and stability of many of the reported organic mixed ionic-electronic conductors.

## Abstract (web summary)

The optimization of organic mixed ionic-electronic conductor is critical to realize high performance organic electrochemical transistors. Here, the authors demonstrate the removal of residual palladium impurities to be the key factor to achieving a figure-of-merit of [μC*] of over 2000 V^−1^ cm^−1^ s^−1^.

## Introduction

Organic mixed ionic-electronic conductors (OMIECs) are materials capable of conducting both ionic and electronic charge carriers, and operate via volumetric-electronic coupling rather than an interfacial regime^1^. These properties enable efficient charge storage and signal transduction, making OMIECs applicable to a plethora of applications, including thermoelectric generators^2,3^, actuators^4,5^, inverters^6^, sensors^7–9^, light-emitting electrochemical cells^10,11^, and batteries^12–14^. OMIECs are also employed as the channel material in organic electrochemical transistors (OECTs) on account of their low operational voltages and ability to transduce and amplify an ionic signal into a readable electronic output^15^. This, combined with the biological compatibility conferred by organic materials, renders OECTs a promising technology for sensing applications in healthcare settings^16^, as well as in neuromorphic computing^17,18^.

Typically, OECTs are comprised of an OMIEC channel, separating a source and a drain electrode, coupled to a gate electrode through an electrolyte (Supplementary Fig. 1). Application of a gate voltage (*V*_G_) drives ions from the electrolyte into the OMIEC. These compensate electronic charges in the channel, which in turn modulates the OMIEC channel conductivity. Channel currents driven by an applied source-drain bias are sensitive to the gate-induced channel conductance modulation, thus allowing for gate signals to be transduced and amplified. This difference between the three-dimensional nature of OECTs and the interfacial operation of organic thin film transistors (OTFTs)^19^ presents new challenges to current understanding and indicates that previous assumptions on the similarity of OTFTs and OECTs may not hold true^3^.

The performance of OECT devices is typically evaluated by examining the steady-state electrical performance, which can be represented by the maximum transconductance (*g*_m_) achieved in devices, calculated from:

1 $${g}_{{{{{{\rm{m}}}}}}}={{{{\mu }}}}{C}^{*}\frac{{Wd}}{L}\left({V}_{{{{{{{\rm{TH}}}}}}}}-{V}_{{{{{{\rm{g}}}}}}}\right)$$

where *μ* is the electronic charge carrier mobility, C* is the volumetric capacitance, *W*, *d*, and *L* are the channel width, depth and length respectively, *V*_TH_ is the threshold voltage and *V*_g_ is the gate voltage^20^. Transconductance is a direct measure of an OECT’s ability to amplify a signal, which can be critical for certain applications, such as in biosensing, where a small ionic fluctuation can be transduced into a larger, readable electronic output. Equation (1) highlights the dependence of *g*_m_ on both material properties (μC*) and device geometry (*Wd*/*L*). As an alternative figure-of-merit to *g*_m_ for understanding the performance of an OECT and to allow for direct material comparisons, the product μC* is commonly employed instead, to eliminate geometry dependence^21^. Other important parameters when considering the application of an OECT include the ON/OFF ratio and the switching speed. The ON/OFF ratio, similar to an OFET, refers to the ratio of the maximum *I*_d_ recorded at a particular *V*_D_, and the *I*_d_ measured in the OFF state. Ideal transistors exhibit a low ON resistance and extremely high OFF resistance, which result in a high ON/OFF ratio. A low number (i.e., a high OFF current) is indicative of current leakage or ambient doping of the semiconductor material. Alternatively, a low mobility (i.e., a low ON current) would also result in a low ON/OFF ratio, thus maximizing the charge carrier mobility is critical for meaningful device applications beyond achieving the highest possible μC*. The switching speed is also critical when considering oscillations in brain activity, for example, which can occur on frequencies in the range of 0.5–200 Hz^15,22^. Currently, OECTs are able to achieve switching speeds in the microsecond timescales.

One factor that has been demonstrated to influence the performance of organic semiconducting polymers is the molecular weight. Typically in the field of organic transistors and molecular weight studies, the number average molecular weight, *M*_n_ is the most widely reported^23–28^. *M*_n_ is defined as the total weight of the polymer divided by the total number of polymer chains, and the dispersity (*Ð*) is a measure of the distribution of the molecular weight within a sample, with a *Ð* of one being optimal and indicating all polymer chains are of equal length. Information on the methods of measuring molecular weight and their respective advantages and limitations are detailed in the Supplementary Information.

Many studies have been carried out on the relationship between charge carrier mobility in OTFTs and the molecular weight of the organic semiconducting layer, arising from mainly morphological factors^29–33^. Less is known about the influence of molecular weight in OECT devices that mostly differ from OTFTs by the swelling and bulk doping of the active layer due to ion penetration/expulsion. The dependence of OTFT performance on the molecular weight of the active semiconductor has typically focused on poly(3-hexylthiophene) (P3HT), where both individual batches and blends of different molecular weights have been examined^30,31,33,34^. Longer polymer chains, occurring in higher molecular weight batches, act as tie chains, connecting crystalline domains and maximizing charge carrier mobilities. This was confirmed by blending low and high molecular weight batches of P3HT in varying percentages, until a plateau was reached in the OTFT mobility^31^. Without the addition of higher molecular weight polymer chains to act as tie chains, the frequency and prominence of the grain boundaries between crystalline regions force charges to move by unfavorable hopping events, severely limiting the charge carrier mobility^29^. Based on OTFT studies, a decrease in the electronic mobility might be expected with lower molecular weight but studies using blended systems demonstrated that more favorable morphology can arise from an intermediate molecular weight^35^. Although this has been shown for ladder-type polymers^36^, conventional OECT materials, such as polythiophene or naphthalenediimide (NDI)-based polymers, have a less rigid structure. Thus, the link between molecular weight of ladder-type polymers and OECT performance cannot be translated directly to other OMIECs and the relationship between OECT performance and OMIEC molecular weight has not been decoupled yet.

Additionally, the purification of OMIEC polymers is rarely considered. The vast majority of OMIEC conjugated polymers are synthesized using Pd-mediated cross-coupling conditions^19,24,25,28,37,38^ and these materials are typically only purified by simple Soxhlet extraction in numerous solvents, which removes limited amounts of residual metal impurities. Specifically, Stille polymerizations make use of around 3000–5000 ppm of Pd, and while Soxhlet extraction can reduce levels by an order of magnitude, this may not be sufficient to maximize performance^39^. These levels have been shown to impact OPV applications^40^, as residual metals, such as palladium (Pd), may act as a charge sink, trapping mobile charges and reducing charge transport properties^39,41^. Alternatively, Pd can act as a co-catalyst to facilitate charge transfer and catalyze redox reactions of ambient species (i.e., in the operation conditions used in OECT but not OTFT applications)^42^, including the oxygen reduction reaction (ORR), which generates toxic hydrogen peroxide, making impure OMIECs unsuitable for bioelectronic applications.

**Fig. 1:**
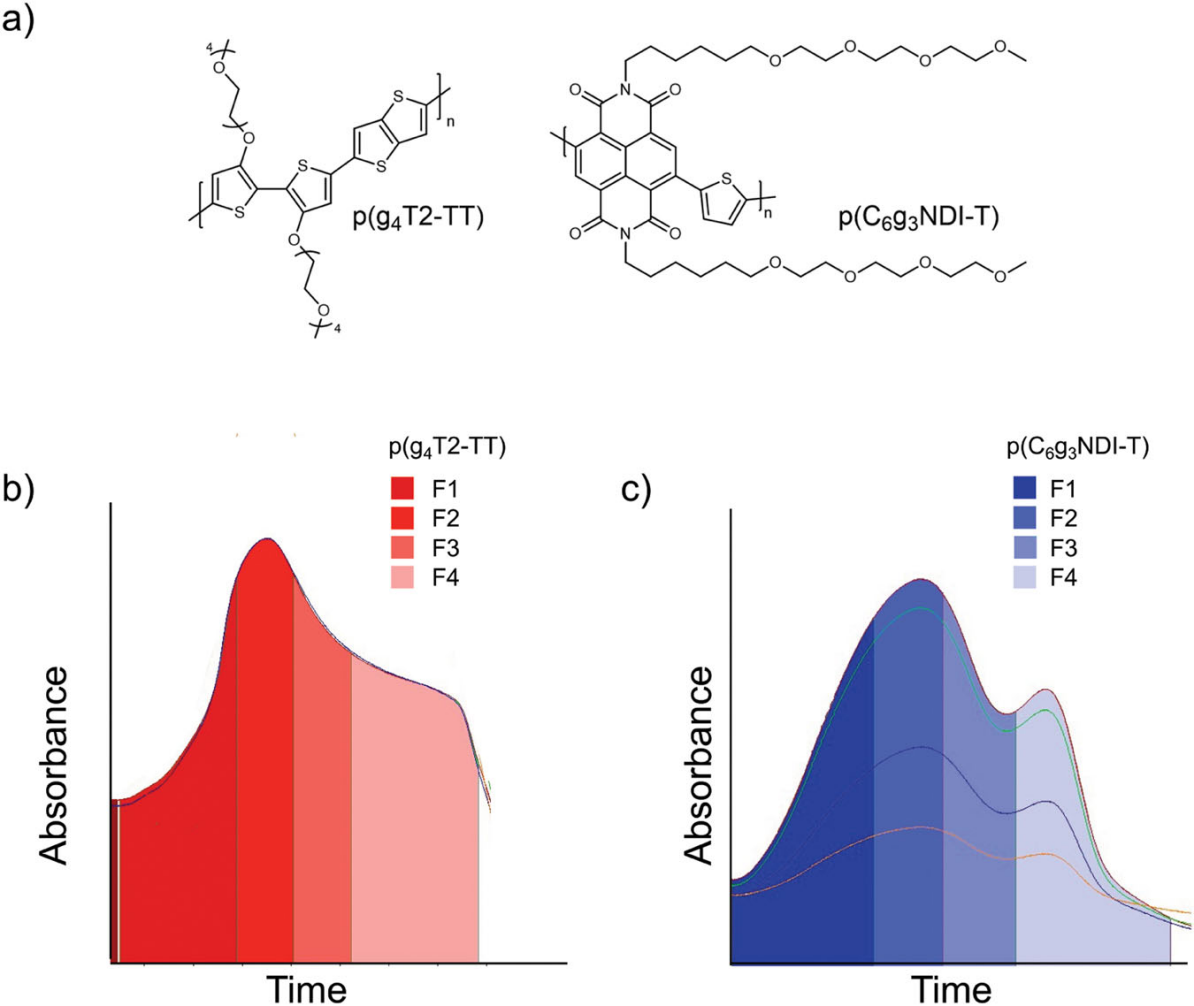
Chemical structures and fractionation traces of the two investigated polymer. **a** Chemical structures of p(g_4_T2-TT) and p(C_6_g_3_NDI-T). The preparative gel permeation chromatography trace for **b** p(g_4_T2-TT) and **c** p(C_6_g_3_NDI-T) showing the second cycle, where the polymer is collected into four separate fractions, F1–4.

Herein, we present an investigation into the impact of purity (the level of residual Pd) and molecular weight of the most common p-type and n-type backbones (polythiophene and NDI, respectively, Fig. 1a) on the OECT performance. These were prepared by standard Stille polymerizations, using 2 mol% Pd catalyst (which equates to ~3000 ppm for p(g_4_T2-TT) and ~4,900 ppm for p(C_6_g_3_NDI-T)), and the different molecular weights were obtained by preparative GPC (Fig. 1b, c), which conveniently offers the ability to narrow *Ð* whilst simultaneously removing residual metal impurities. The optoelectronic properties, OECT performance, and structural properties using grazing incidence wide-angle X-ray scattering (GIWAXS) of four molecular weight fractions of *Ð* < 2 (Table 1) of polymers p(g_4_T2-TT) and p(C_6_g_3_NDI-T) were studied and compared to the unpurified polymer (i.e., the polymer after standard Soxhlet procedure but pre-fractionation). In particular, the impact of purity on the OECT performance was probed (Fig. 1).

This study utilizes an alternative method for OMIEC purification, by more thoroughly removing by-products than only using traditional Soxhlet purification. Preparative GPC is also particularly useful for studying the impact of molecular weight as any batch-to-batch variations are removed. One batch is simply divided into several fractions of lower dispersity instead of synthesizing new batches through different reaction conditions, making this the most comprehensive method for comparing molecular weights.

## Results and discussion

### Material synthesis

The synthetic routes to p(g_4_T2-TT) and p(C_6_g_3_NDI-T) are shown in Supplementary Figs. 2–5. Monomer 13,13’-((5,5’-dibromo-[2,2’-bithiophene]-3,3’-diyl)bis(oxy))bis(2,5,8,11-tetraoxatridecane) (g_4_T2Br_2_) was synthesized through a modified literature procedure, by utilizing a copper(I) iodide catalyst to decorate the 3,3’-dibromo-2,2’-bithiophene core with tetraethylene glycolated side chains, followed by a typical bromination using *N*-bromosuccinimide to generate the monomer in a 56% yield (Supplementary Fig. 6)^43^. Alternatively, 4,9-dibromo-2,7-di(2,5,8,11-tetraoxaheptadecan-17-yl)benzo[lmn][3,8]phenanthroline-1,3,6,8(2H,7H)-tetraone (C_6_g_3_NDI-Br_2_) was prepared from 2,6-dibromonaphthalene-1,4,5,8-tetracarboxylic dianhydride (NDA-Br_2_) using a single step procedure, by treating NDA-Br_2_ with 2,5,8,11-tetraoxaheptadecan-17-amine (Supplementary Fig. 7)^24^. Lastly, both p(g_4_T2-TT) and p(C_6_g_3_NDI-T) were synthesized via Stille polymerization reactions of g_4_T2Br_2_ or C_6_g_3_NDI-Br_2_ respectively, with equimolar quantities of the appropriate stannylated thiophene derivative (Supplementary Figs. 8 and 9). Each polymer was initially purified using the traditional Soxhlet purification method. While this has been documented as a method for separating molecular weights^44^, the disadvantage is that fractions eluting later will likely have a higher purity than those eluted first. Furthermore, none of these polymers were soluble in the Soxhlet solvents used prior to chloroform, so the dispersity could not be reduced by this method. Instead, we employed preparative GPC to separate a single batch of polymer into four molecular weight fractions, such that each fraction contained approximately the same amount of material, with the first fraction collected (fraction 1) being of highest molecular weight (Fig. 1b, c). Preparative GPC can also be viewed as an additional method of purification, as remaining impurities (typically Pd from the catalyst) are of different molecular weight than any polymer chains, thus are discarded before or after the elution of the polymer.

### Materials’ properties

Analytical GPC was employed to estimate the molecular weights of the two series of polymers and their weight fractions, with dimethylformamide and chloroform as eluent for p(g_4_T2-TT) and p(C_6_g_3_NDI-T), respectively. The measurements were conducted at 40 °C with respect to polystyrene standards. The values obtained are summarized in Table 1. It should be noted that despite glycolated polymers often exhibiting a bimodal distribution from GPC^19,25^, these polymers, particularly once fractionated, showed ideal unimodal traces (Supplementary Figs. 10–12). This is expected, as the preparative GPC used for fractionation relies upon the same size exclusion method as the analytical GPC.

**Table 1:** Summary of the two polymer series’ physical and optoelectronic properties

Polymer		*M*_n_ (kDa) [*Ð*]^a^	*E*_g,opt_ (eV)^b^	*λ*_max, sol_ (nm)^c^	EA (eV)^d^	IP (eV)^d^
p(g_4_T2-TT)	Unpurified	83.3 [1.36]	1.67	610	–	4.23
	F1	180.1 [1.30]			–	4.24
	F2	107.9 [1.12]			–	4.25
	F3	61.3 [1.25]			–	4.24
	F4	18.4 [1.15]			–	4.26
	Recombined	90.6 [1.28]				
p(C_6_g_3_NDI-T)	Unpurified	42.4 [2.13]	1.65	323, 540	4.19	–
	F1	211.0 [1.95]			4.18	–
	F2	99.8 [1.34]			4.18	–
	F3	46.2 [1.67]			4.17	–
	F4	17.6 [1.48]			4.19	–
	Recombined	61.2 [1.92]				

^a^Number-average molecular weight (*M*_n_) and dispersity (*Ð*) measured by GPC vs polystyrene standards in dimethylformamide for p(g_4_T2-TT) or chloroform for p(C_6_g_3_NDI-T) at 40 °C.

^b^Optical band gap estimated from thin film UV-Vis absorption onset.

^c^Peak wavelength obtained from solution UV-Vis absorption spectra in chloroform.

^d^Calculated from the onset of the cyclic voltammogram of polymer thin films on glassy carbon electrode with 0.1 M tetrabutylammonium hexafluorophosphate as the supporting electrolyte using a platinum counter electrode and a silver/silver chloride reference electrode (Supplementary Fig. 13).

The two series’ optoelectronic properties are summarized in Table 1. Cyclic voltammetry (CV) in 0.1 M tetrabutylammonium hexafluorophosphate (TBAPF_6_) in acetonitrile was used to determine the ionization potential (IP) of p(g_4_T2-TT), which remained unchanged across the fractions at ~4.25 eV, providing an estimate for the highest occupied molecular orbital (HOMO) (Supplementary Fig. 13a)^45^. The electron affinity (EA) of p(C_6_g_3_NDI-T) also showed no significant variation at ~4.18 eV and provided an estimate for the lowest unoccupied molecular orbital (LUMO) (Supplementary Fig. 13b). Each polymer exhibited partly reversible discrete reduction or oxidation behavior. Due to the relatively narrow electrochemical stability window of the electrolyte, the EA for p(g_4_T2-TT) and IP for p(C_6_g_3_NDI-T) could not be accessed using this method.

The UV-Vis absorption spectra of the polymers in solution and as thin films are presented in Supplementary Figs. 14 and 15, respectively. p(g_4_T2-TT) showed a characteristic absorption profile as seen in previous reports of similar polymers^46^, where the *λ*_max_ (≈600 nm) is attributed to the π–π* transition (HOMO-LUMO transition), while the small lower energy band (≈950 nm) is most likely attributed to ambient-induced polarons. As observed previously, both bands for p(C_6_g_3_NDI-T) were blue-shifted to higher energy compared to p(g_4_T2-TT), with the π–π* transition ≈345 nm and the peak ≈600 nm attributed to the intramolecular charge transfer (ICT) from the electron-rich bithiophene units to the electron-deficient NDI units^24,47,48^. In the UV-Vis absorbance spectra in solution of p(g_4_T2-TT) (Supplementary Fig. 14a), no band at ∼950 nm is observed, confirming its attribution to doping by ambient species in the solid-state. The normalized UV-Vis data supported the uniformity noted by CV for each polymer series, providing almost identical spectra across the molecular weights, which is unsurprising, given that no impact on the bandgap is expected above the effective conjugation limit. The subtle differences observed in the vibrionic structure for p(g_4_T2-TT) and the shift of the ICT band for p(C_6_g_3_NDI-T) indicated that different packing may be induced by the change in molecular weight (more clearly indicated in Supplementary Information, Supplementary Fig. 16).

Inductively coupled plasma mass spectrometry (ICP-MS) was employed to calculate the concentration of residual palladium contaminants in each fraction (Table 2), which originates from the Pd-based catalysts required for Stille polymerizations. In both polymers, the levels of palladium observed before preparative GPC fractionation were on the same order of magnitude, at 1769 and 1176 ppm, for the p-type and n-type polymers, respectively. For both OMIEC polymers, this data demonstrated the impact of preparative GPC fractionation on purity, whereby all fractions contained less Pd than the unpurified fractions, with a decrease of between 54 and 97% in Pd levels observed. This translated to Pd levels as low as 39 and 73 ppm, validating preparative GPC as a consistent method for purification.

**Table 2:** Steady-state OECT performance of polymers

Polymer		Conc. of Pd [ppm]^a^	*µ*_OECT_ [cm^2^ V^−1^ s^−1^]^b^	C* [F cm^−3^]^c^	[μC*] [F V^−1^ cm^−1^ s^−1^]^d^	*g*_m_*’* [S cm^−1^]^e^
p(g_4_T2-TT)	Unpurified	1769	3.13 ± 0.52	120 ± 9	374 ± 67	295 ± 57
	F2	370	5.31 ± 2.65	246 ± 21	1308 ± 662	838 ± 438
	F1	323	5.65 ± 0.19	123 ± 15	694 ± 87	494 ± 17
	F3	157	6.53 ± 0.065	308 ± 20	2008 ± 130	1457 ± 6
	F4	73	8.53 ± 0.40	114 ± 14	972 ± 127	626 ± 38
	F3 + Pd_2_(dba)_3_	2157	3.02 ± 0.26	287 ± 18	860 ± 56	602 ± 21
	Recombined^f^	236	4.75 ± 1.12	123 ± 17	451 ± 124	231 ± 56
p(C_6_g_3_NDI-T)	Unpurified	1176	1.02 × 10^−^^3^ ± 3.79 × 10^−^^4^	222 ± 79	0.227 ± 0.117	0.055 ± 0.021
	F4	540	2.26 × 10^−^^3^ ± 2.53 × 10^−^^4^	250 ± 78	0.563 ± 0.188	0.164 ± 0.018
	F1	197	3.57 × 10^−^^3^ ± 2.94 × 10^−^^4^	129 ± 55	0.461 ± 0.202	0.127 ± 0.017
	F3	112	6.06 × 10^−^^4^ ± 1.58 × 10^−^^4^	521 ± 160	0.316 ± 0.127	0.095 ± 0.024
	F2	39	5.34 × 10^−^^3^ ± 3.40 × 10^−^^4^	306 ± 127	1.632 ± 0.688	0.155 ± 0.020
	F2 + Pd_2_(dba)_3_	2039	1.10 × 10^−^^3^ ± 1.52 × 10^−^^4^	124 ± 26	0.136 ± 0.0341	0.029 ± 0.003
	Recombined^f^	198	1.96 × 10^−^^3^ ± 2.25 × 10^−^^4^	257 ± 101	0.503 ± 0.205	0.110 ± 0.012

^a^Calculated from inductively coupled plasma mass spectrometry (ICP-MS, detailed in the SI).

^b^OECT mobility calculated from the transistor saturation µC* product using the respective C* values.

^c^C* values extracted from Randles circuit fits from electrochemical impedance curves of the polymers coated on Au electrodes in 0.1 M aqueous NaCl solution.

^d^[μC*] calculated for the highest performing channel from the slope of *g*_*m*_ with known channel dimensions and applied bias.

^e^Peak OECT transconductance normalized by thickness.

^f^Formed by combining a mass ratio of 1:1.32:0.16:1 of F1:F2:F3:F4 for p(g_4_T2-TT), which resulted in *M*_n_ = 90.6 kDa and a mass ratio of 1:1.4:1.4:1 of F1:F2:F3:F4 for p(C_6_g_3_NDI-T), which resulted in *M*_n_ = 61.2 kDa. All measurements are an average across 6 devices.

**Fig. 2:**
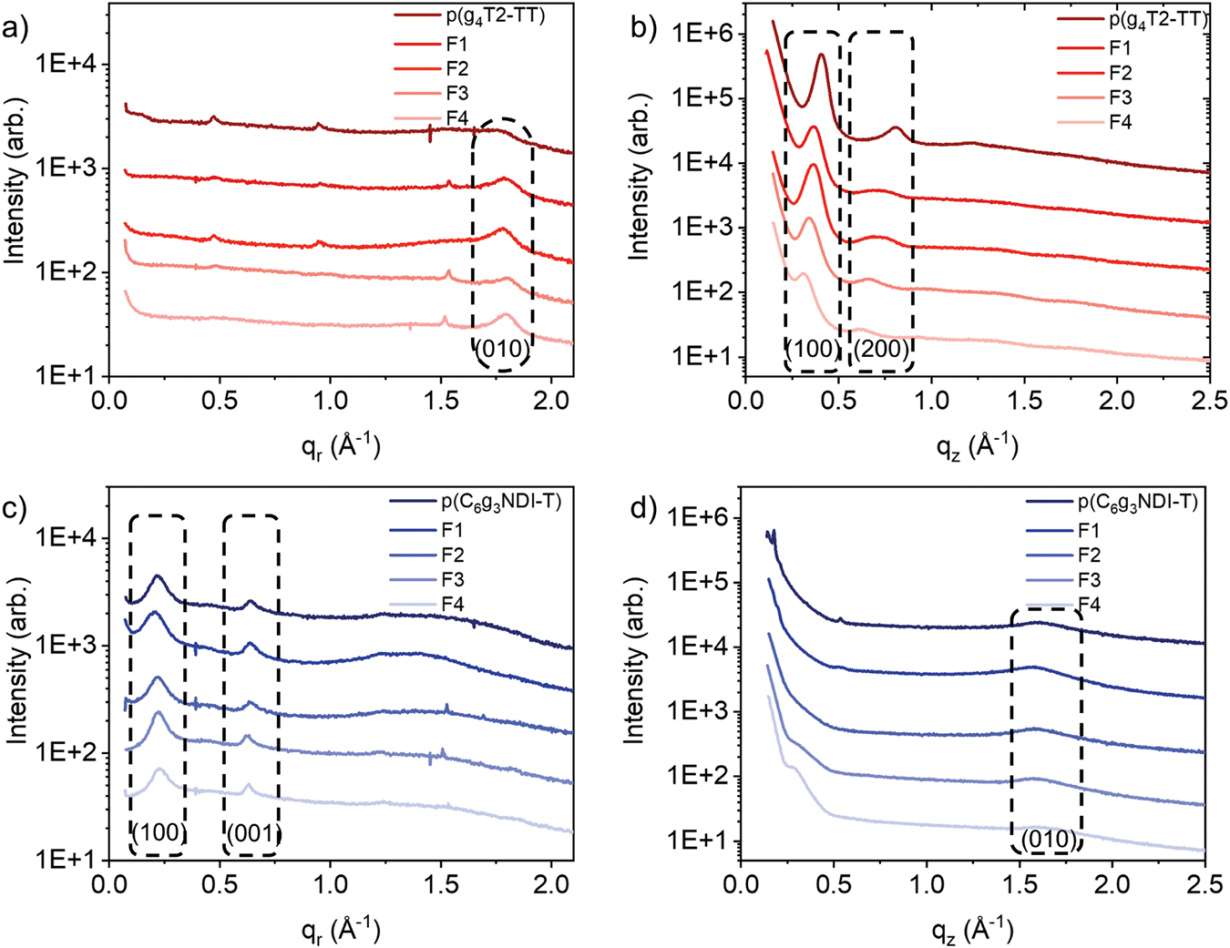
Two-dimensional grazing incidence wide angle X-ray scattering. GIWAXS line cuts of as-cast thin films of the unpurified and four fractions of the two polymers. showing **a** in-plane (*q*_*r*_) and **b** out-of-plane (*q*_*z*_) line cuts of each fraction of p(g_4_T2-TT) and **c** in-plane and **d** out-of-plane line cuts of each fraction of p(C_6_g_3_NDI-T).

To investigate the impact of fractionation (reduced dispersity and reduced concentration of Pd) on the polymer microstructure, grazing incidence wide-angle X-ray scattering (GIWAXS) measurements were carried out. The line-cuts of the as-spun thin films are displayed in Fig. 2 and the 2D GIWAXS patterns and peak parameters are included in the Supplementary Information (Supplementary Figs. 17 and 18). p(g_4_T2-TT) compared well to previously reported glycolated polythiophene derivatives^28,43^, presenting an edge-on orientation. To quantitatively interpret the GIWAXS data, pseudo-Voigt peak fits were used to extract disorder associated coherence lengths in the backbone and lamellar directions. The unpurified p-type polymer had a poorly resolved π-stack compared to the four fractions, which may be because of the larger *Ð* (Table 1). Notably, after fractionation, the in-plane π-stack contracted by 0.05–0.09 Å and displayed longer coherence lengths (narrower diffraction peaks) in the backbone direction compared to the unpurified polymer (Supplementary Fig. 17). p(g_4_T2-TT) showed evidence of two orders of out-of-plane lamellar scattering, with the fractions showing expanded lamellar spacing and a reduction in the coherence length in the lamellar direction of 10–25%.

Contrastingly, p(C_6_g_3_NDI-T) showed a face-on orientation, in agreement with previously reported NDI polymers^19,49^. The (010) out-of-plane π-stack scattering displayed broad and relatively weak peaks due to scattering arising from a small number of chains stacked together, which can usually be attributed to a high degree of paracrystalline disorder^50^. A trend in π-π stacking distance was observed, with a small expansion in the out-of-plane *d*-spacing with increasing *M*_*n*_. This also corresponds to the bathochromic shift observed in the ICT band in the UV-Vis absorption spectra with decreasing molecular weight, indicating subtle differences in packing (Supplementary Fig. 16b). Previous results have shown that for NDI-T2 backbones, two different packing configurations are possible, namely Form I and Form II^51^. Form I occurs where the NDI motif aligns with an NDI from a neighboring chain through cofacial registration, resulting in double the backbone periodicity, whilst in a Form II configuration the NDI unit aligns with a bithiophene unit^51^. For p(C_6_g_3_NDI-T), the (001) in-plane backbone spacing of 19.6–20.0 Å was consistent with the length of an NDI-T repeat length, thus it can be assumed that predominantly Form I stacking was present.

**Fig. 3:**
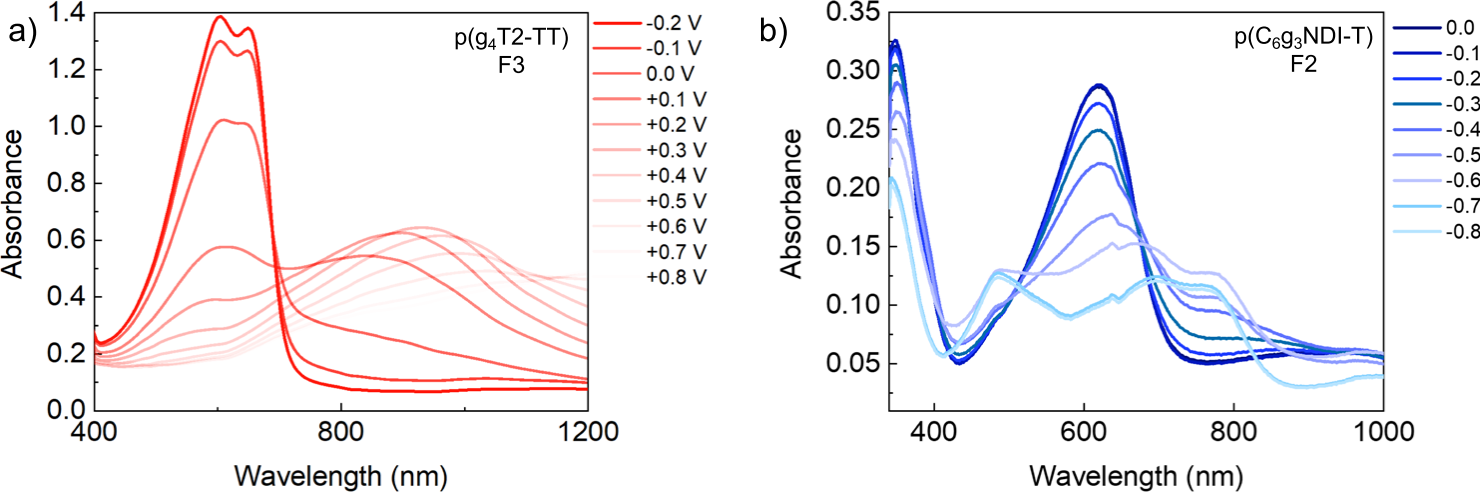
Spectroelectrochemical measurements for the highest performing fraction of each polymer. UV-Vis absorption measurements recorded whilst applying a voltage vs Ag/AgCl using a 0.1 M NaCl aqueous degassed electrolyte, with potential steps of 0.1 V. **a** Fraction 3 of p(g_4_T2-TT) between −0.2 and +0.8 V vs Ag/AgCl and **b** fraction 2 of p(C_6_g_3_NDI-T) between 0 and −0.8 V. The unpurified and other fractions for each polymer are shown in Supplementary Fig. 20.

The electrochemical activity of both series of polymers was further investigated in 0.1 M NaCl aqueous electrolyte through CV and spectroelectrochemical measurements. Aqueous CV (Supplementary Fig. 19) indicated that both polymers at all molecular weights were reversibly doped and de-doped. All fractions of p(g_4_T2-TT) showed comparable oxidation onset potentials of ≈−0.1 V versus Ag/AgCl. The same is true for p(C_6_g_3_NDI-T), where similar reduction onsets of ≈−0.1 V versus Ag/AgCl were recorded. Their doping mechanisms were further explored by spectroelectrochemical measurements, conducted in the same electrolyte. As shown in Fig. 3, Fig. 2a and Supplementary Figs. 20a and 21a, all fractions of p(g_4_T2-TT) were partially doped under ambient conditions and require a negative potential to fully de-dope the films, as indicated by the negative oxidation onset potential. Progressive charging to +0.2 V versus Ag/AgCl showed a gradual bleaching of the π-π* peak, with a simultaneous appearance of a new absorption peak at ≈950 nm, which was attributed to the formation of the polymer’s polaron, as described in previous literature^28,52^. Beyond +0.3 V, this peak began to decrease in intensity and a new peak at higher wavelengths appeared, which is ascribed to the formation of bipolaronic species. Finally, the applied voltage was returned to the initial value of −0.2 V and all fractions of the polymer resumed their original de-doped UV-Vis absorption spectra, indicating good reversibility, with no polymer degradation, in aqueous media.

The n-type polymer, p(C_6_g_3_NDI-T), was charged between 0.0 and −0.8 V versus Ag/AgCl (Fig. 3, Fig. 2b, and Supplementary Figs. 20b and 21b), and all fractions showed a fully de-doped state at 0 V. Generally, by charging at −0.8 V, the ICT band intensity decreases, while two new absorption features appear at ≈480 nm and ≈790 nm. They are assigned to transitions to the S1 and S2 states, which are dominated by π-π* and ICT, as suggested by literature^47^. Notably, all fractions displayed a large doping extent, which was reversible, as shown by a return to the fully de-doped polymer UV-Vis absorption spectrum when charging is returned to the initial value. From the consistent UV, CV and spectroelectrochemical data across all fractions of the two polymers, it was concluded that the degree of doping remains constant, and not dependent on the molecular weight or the degree of purity in both cases.

### Organic electrochemical transistor performance

OECT devices were fabricated and the electrical performance measured for each fraction, in order to elucidate the impact of the palladium. The devices were fabricated according to the procedure described in the Methods section, with a device geometry of *W* = 100 µm and *L* = 10 µm. All devices were operated in accumulation mode, and data was obtained without any post-processing treatments (see Supplementary Figs. 22–25 for output, transfer and transconductance curves) and Table 2 summarizes the OECT parameters.

The impact of Pd impurities on the OECT performance was firstly evaluated by comparing the Pd concentrations in each fraction, measured by ICP-MS, to the charge carrier mobilities (Table 2). Across F1-4 of both materials, where Pd concentrations are reduced, the charge carrier mobility is increased in seven out of eight cases (Table 2). We propose that the origins of these observations arise from the Pd(0) nanoclusters, formed from the molecular Pd cross-coupling catalyst during the polymerization reactions^40,53,54^. Such species may be able to act as catalysts for ORR due to the favorable energetic offsets^55^. For example, the LUMO of p(C_6_g_3_NDI-T) has an energy of approximately −4.18 eV, considerably higher than the −5.1 to −5.2 eV work function of Pd^56,57^, meaning that both the electron transfer from the polymer electron polaron to Pd and the ORR itself are energetically favorable, leading to polaron annihilation. This mechanism of charge transfer explains why the n-type polymer observes a significant increase in mobility upon the removal of Pd. When mobile electrons are transferred to the Pd(0) nanoclusters to catalyze the ORR, the electron mobility is reduced. It should be noted that this reaction is only possible in aqueous conditions, such as those used in OECT operation. Additionally, ORR can also generate hydrogen peroxide, which is biologically toxic, hindering any application as a safe bioelectronic sensor^58^. Interestingly the mobility of the p-type material was observed to be negatively affected by higher palladium levels, albeit by a smaller factor than the n-type (twofold decrease compared to fivefold). There are no accessible oxidation reactions in this case that could be driven by the hole polaron energies. Impedance measurements on p-type polymers with similar energy levels to p(g_4_T2-TT) have previously indicated that increased Pd content can cause increased electrical resistance^41^. It is possible that the presence of Pd nanoclusters in the OECT polymer thin films creates local distortions in the semiconductor microstructure, and a subsequent increase in energetic disorder, leading to a reduction in mobility observed^40,41,59^.

**Fig. 4:**
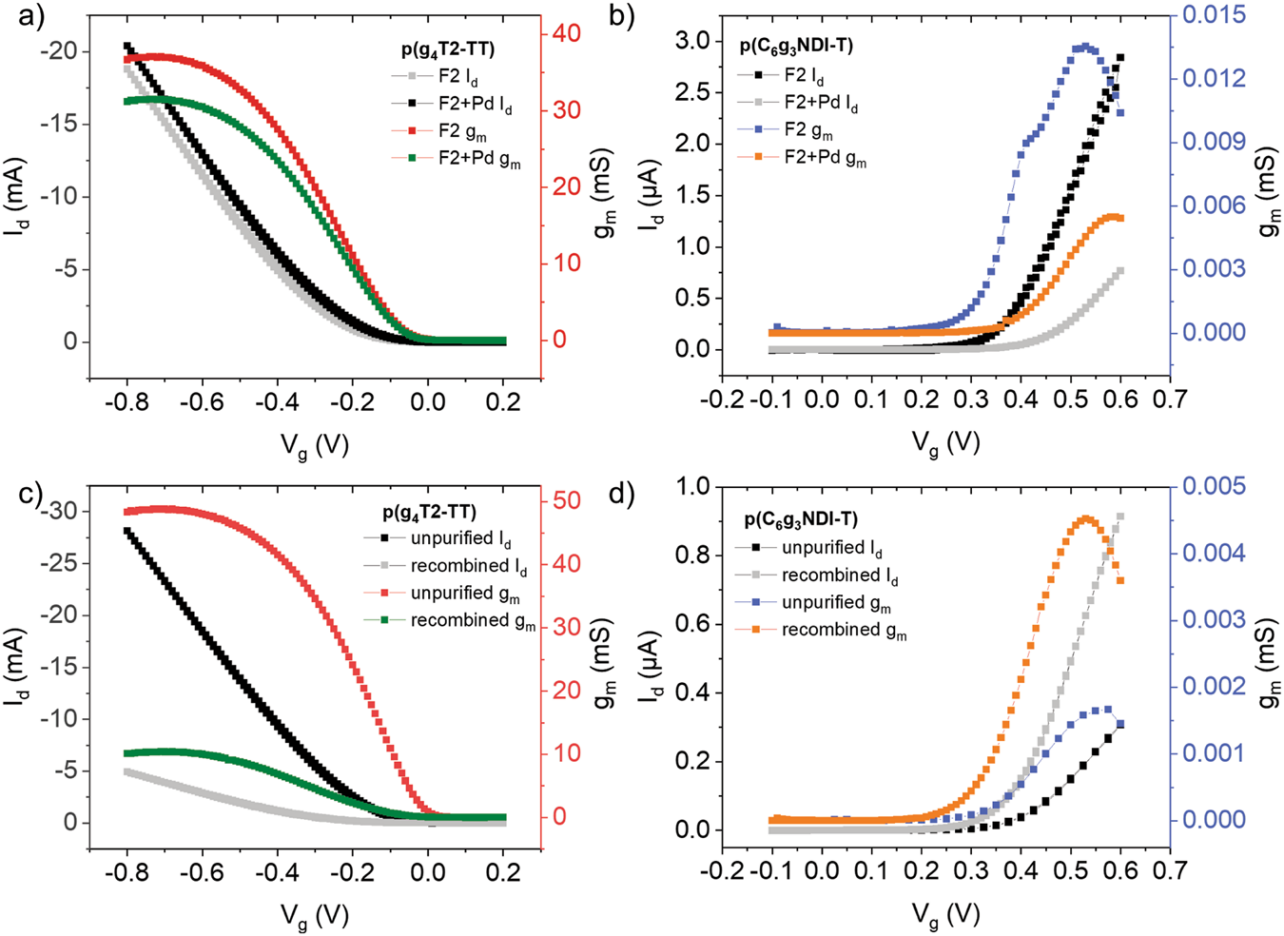
OECT transfer and transconductance characteristics before and after the reintroduction of Pd and comparing the unpurified and recombined batches of each polymer. The OECT transfer and transconductance characteristics for **a** fraction 3 of p(g_4_T2-TT) before and after the reintroduction of 2000 ppm of Pd and **b** fraction 2 of p(C_6_g_3_NDI-T) before and after the reintroduction of 2000 ppm of Pd. Transfer and transconductance characteristics for **c** unpurified and recombined p(g_4_T2-TT) and **d** unpurified and recombined p(C_6_g_3_NDI-T). All measurements are an average across 6 devices. *I*_d_ refers to the drain current, *V*_g_ is the gate voltage and *g*_m_ is the transconductance.

In addition to containing differing concentrations of palladium, each sample however also differed in both molecular weight and dispersity. To decouple the influence of these parameters on the OECT charge carrier mobilities, all GPC fractions for each polymer were recombined and the OECT characteristics were measured (see recombined rows in Table 2, Fig. 4c, d, and Supplementary Figs. 26 and 27). In this way, any effects arising from the molecular weight variations or reduced dispersities of the fractionated batches are eliminated. Furthermore, the physical form of Pd is representative of that present in the unpurified polymers (i.e., nanoclusters vs molecular Pd). The impact of purification appears to offer a greater improvement for p(C_6_g_3_NDI-T) than p(g_4_T2-TT). More specifically, there is a 92% increase in electron mobility from 1.02 × 10^−^^3^ to 1.96 × 10^−^^3^ cm^2^ V^−1^ s^−1^ for p(C_6_g_3_NDI-T), compared to ~50% increase in hole mobility for p(g_4_T2-TT), from 3.13 to 4.75 cm^2^ V^−1^ s^−1^, where both have comparable levels of Pd, at around 200 ppm. The recombined batches of both polymers show comparable C* values to the unpurified batches, resulting in increases to μC* from 0.227 to 0.503 F V^−1^ cm^−1^ s^−1^ for p(C_6_g_3_NDI-T) and from 374 to 451 F V^−1^ cm^−1^ s^−1^ for p(g_4_T2-TT). Notably the recombined batches of both polymers show a lower performance than most of the fractions. This can be explained by the GIWAXS data (Fig. 2 and Supplementary Figs. 17 and 18), where fractionation resulted in both a decrease in π-π stacking distance and an increase in coherence length, both of which would be expected to result in an increase in charge carrier mobility.

To further explore the causal relationship between the concentration of Pd and the OECT performance, we reintroduced 2000 ppm of Pd back into the highest performing fraction, in the form of the Pd_2_(dba)_3_ catalyst. The OECT characteristics were remeasured (Fig. 4a, b). For fraction 3 of p(g_4_T2-TT), this resulted in lower performance, with hole mobility decreasing from 6.53 to 3.02 cm^2^ V^−1^ s^−1^ (Fig. 4a and Supplementary Fig. 28). For fraction 2 of p(C_6_g_3_NDI-T), the reintroduction of Pd also resulted in the mobility decreasing from 5.34 × 10^−^^3^ to 1.10 × 10^−^^3^ cm^2^ V^−1^ s^−1^ (Fig. 4b and Supplementary Fig. 29). The p-type polymer observed a smaller decrease than the n-type polymer, which is consistent with the relative differences observed between the unpurified and remixed sample measurements.

Ionic impurities in thienothiophene-based polymers have previously been shown to also have little impact on charge carrier mobility in OFET devices^60^. To explore this correlation in our polymers, the unpurified and highest OECT performance fraction of each polymer were tested in a top gate/bottom contact OTFT and compared, (the output and transfer characteristics for which can found in Supplementary Figs. 30 and 31). The n-type polymer, p(C_6_g_3_NDI-T), displayed low output currents, which resulted in poor electron mobilities of 1.7 × 10^−^^4^ and 6.0 × 10^−^^5^ cm^2^ V^−1^ s^−1^ for the unpurified and fraction 2, respectively. Interestingly, the unpurified batch exhibits an order of magnitude greater electron mobility than the fractionated batch, supporting previous observations that material purity has little impact on OTFT performance. Instead, it is possible that the higher molecular weights present in the unpurified batch led to more favorable morphology, which is a dominant effect for OTFT performance. The hole transporting polymer, p(g_4_T2-TT), displayed conducting properties with no current modulation, so no mobility was able to be extracted.

**Fig. 5:**
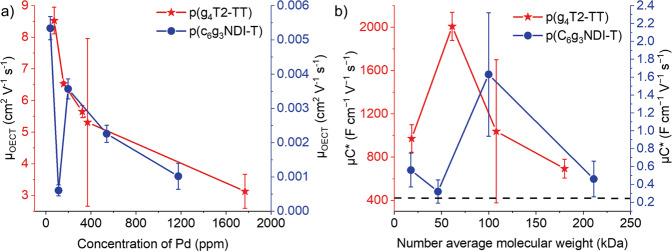
Comparing concentration of Pd and the molecular weight with the OECT performance. **a** *μ*_OECT_ as a function of Pd content in ppm for p(g_4_T2-TT) and p(C_6_g_3_NDI-T) and **b** [μC*] as a function of *M*_n_ for p(g_4_T2-TT) and p(C_6_g_3_NDI-T), whereby the dashed line indicates the performance of the polymer pre-fractionation. The error bars are the standard deviation of the data set and *µ*_OECT_ is the calculated OECT mobility.

For the p(g_4_T2-TT) series, the thickness normalized transconductance increased to a peak of 1457 S cm^−1^ for fraction 3 (concentration of Pd = 157 ppm), then decreased as the *M*_n_ further increases. A similar trend is observed for p(C_6_g_3_NDI-T), where the highest normalized transconductance was observed for fraction 2 (concentration of Pd = 39 ppm), at 0.155 S cm^−1^. Interestingly, for both series, all values of the fractions were larger than that of the unfractionated batch, which is likely to be caused by the increased charge carrier mobility. [μC*] was calculated using Eq. (1), where the same trends were observed, with the p-type increasing from 694 to 1308 to 2008 F V^−1^ cm^−1^ s^−1^, a very high OECT performance^23,28,61,62^, then decreasing to 972 F V^−1^ cm^−1^ s^−1^ (Fig. 5a). p(C_6_g_3_NDI-T) also mimicked the thickness normalized transconductance, with fraction 2 (the lowest concentration of Pd, at 39 ppm) providing the maximal [μC*] value at a polymer-best 1.632 F V^−1^ cm^−1^ s^−1^ (Fig. 5b).

These trends were further probed by performing electrochemical impedance spectroscopy (EIS) to determine the volumetric capacitance at offset potentials of 0 to −0.8 V and 0.3 to 0.8 V against Ag/AgCl, for p- and n-type devices, respectively, with a 0.1 M NaCl_(aq)_ electrolyte (see Supplementary Figs. 32 and 33). For p(g_4_T2-TT), C* increased from 114 F cm^−3^ to reach a peak for fraction 3 of 308 F cm^−3^, before decreasing by ~60%. A similar observation was made for p(C_6_g_3_T2-T), where again fraction 3 provides the optimal C* of 521 F cm^−3^. Both trends were supported by the doping efficiency seen by spectroelectrochemical measurements, whereby the π-π* transition in each case was normalized to one and the peak associated with the polaron was plotted against each voltage (Supplementary Fig. 21).

It is expected that this trend is observed due to the microstructure of fraction 3 providing the optimal arrangement to promote ionic miscibility^3^. As it is known that the microstructure is influenced by the molecular weight^31,33^, and it is therefore suggested that the molecular weight of fraction 3 is optimal for a high C* value. As documented in literature, a high C* value may indicate that whilst a polymer is able to accommodate compensatory ions, this results in a disruption to the microstructure, leading to a decreased mobility^28,63^. This can be partially observed for both polymer series, for example, fraction 3 of p(C_6_g_3_NDI-T) had the highest C* value, but the lowest *μ*_OECT_ despite lower concentration of electron trapping Pd. This demonstrates that a fine balance between *μ*_OECT_ and C* is required to maximize OMIEC performance in OECTs, which can evidently be influenced by polymer molecular weight. Whilst molecular weight may to some extent impact OECT performance (evaluated by *g*_m_ and [μC*]), both polymer series indicate that the palladium concentration has more influence.

**Fig. 6:**
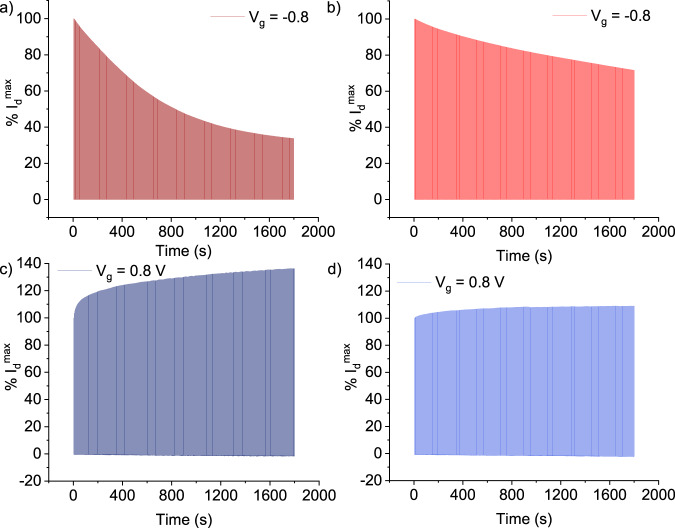
OECT stability measurements in 0.1 M NaCl aqueous electrolyte. Probing stability by cycling *V*_g_ and recording *I*_d_ over 30 min. The graphs indicate the percentage decrease in drain current (*I*_d_) for **a** unpurified p(g_4_T2-TT), **b** F2 of p(g_4_T2-TT), **c** unpurified p(C_6_g_3_NDI-T), and **d** F2 of p(C_6_g_3_NDI-T).

Another critical aspect of OECT performance is the operational stability of devices. Device stability was measured by cycling the gate potential and recording the drain current over 30 min. Generally, it was observed that the stability of the polymer was improved when the Pd content was reduced, with the fractionated materials exhibiting better stability than the unpurified batches (Supplementary Figs. 34 and 35). Numerically, this translates to a change in drain current of 7% for one of the purified fractions of p(C_6_g_3_NDI-T), compared to a 36% change for the unpurified batch (Fig. 6). Similarly, the stability of the p-type polymer improved from a 66% change in drain current for the unpurified batch, to a change of 28% for fraction 2 (Fig. 6). This generates hydrogen peroxide in a two-electron process, which is likely detrimental to OECT performance and can cause degradation of both the OMIEC and the electrodes^58,64^. This therefore indicates that the purity of the polymer plays a role in determining both OECT performance and stability.

To summarize, this study explores the influence of polymer purity and molecular weight on the two most common polymer backbones for OECT applications, a p-type polythiophene and an n-type NDI material. These materials employed preparative GPC as a purification technique for polymers, fractionating a single polymer batch into four batches. By studying the optoelectronic properties using UV-Vis absorption, CV and spectroelectrochemical measurements, the structural properties using GIWAXS and the OECT characteristics, the impact of OMIEC purity on the OECT performance of polymers p(g_4_T2-TT) and p(C_6_g_3_NDI-T) was examined.

To better understand the trend in performance observed across a range of palladium concentrations, particularly the charge carrier mobility, OECT devices were fabricated and measured. We found that the reduced palladium significantly improved the OECT performance, arising mainly from an increase in mobility (of ~60% and 80% for the p- and n-type, respectively), when comparing the unpurified fractions with the purified fractions. We hypothesize that residual Pd arranges in nanoclusters, which act to trap charges and as a co-catalyst for ORR, reducing OMIEC stability. These findings were further verified by reintroducing 2,000 ppm of Pd into the highest performing fraction of each polymer, which saw significant decreases in the OECT mobilities.

These observations highlight the importance of synthetic purification of any semiconducting polymer to be employed in an ambient, aqueous environment. For a valid comparison between OMIECs and to derive accurate structure-property relationships, this work indicates that Pd levels must be as low as possible. As this observation applies to both p- and n-type materials, proper purification of new and existing OMIECs will enhance understanding of OMIECs and improve OECT performance, and preparative GPC offers a versatile method to achieve this.

## Methods

### Materials’ characterization

Column chromatography was performed on silica gel from VWR Scientific, using the indicated solvents. Thin layer chromatography was performed on Silicagel 60 F254 plates purchased from Merck. ^1^H and ^13^C NMR spectra were recorded on a Bruker AV-400 spectrometer at 298 K, with chemical shifts (*δ*) are expressed in parts per million (ppm) downfield from tetramethylsilane (TMS). Deuterated solvents were purchased from Sigma Aldrich. Electrospray (ES-ToF) mass spectrometry was performed using a Micromass LCT Premier. Recycling preparative HPLC was performed using a LaboACE LC-5060 instrument with a separation line containing a 4HH column. The polymers were injected as 5 mg mL^−1^ solutions in chloroform and collected at intervals such that each fraction was approximately one quarter of the material injected, then the solvent removed in vacuo. For OECT characteristics where palladium was reintroduced to the best performing fractions, 2,000 ppm of Pd_2_(dba)_3_ was dissolved in 5 mg mL^−1^ solution of the polymer in chloroform. For OECT characteristics where fractions 1–4 were recombined, this was done so in a mass ratio of 1:1.4:1.4:1 for F1:F2:F3:F4 in order to replicate the original molecular weight profile, and solutions of 5 mg mL^−1^ were used for spin coating the devices. For measurements requiring thin films (UV-vis absorption, spectroelectrochemical measurements, grazing incidence wide-angle X-ray scattering, electrochemical impedance spectroscopy and transistor properties), these were formed by spin coating 50 µL of 5 mg mL^−1^ polymer solutions onto cleaned glass, silicon wafers, ITO or transistor substrates and allowed to air dry. Grazing incidence wide-angle X-ray scattering (GIWAXS) was conducted at the Advanced Photon Source (APS) at Argonne National Laboratory using an incident beam energy of 10.92 keV and an incident angle of 0.14°. Raw scattering data were processed using GIXSGUI^65^ and analyzed using a custom MATLAB script. For inductively coupled plasma mass spectrometry (ICP-MS) measurements, element concentrations were determined based on dry polymer weight. Samples were digested in TraceMetal® grade concentrated nitric acid under elevated temperature (200 °C) and pressure in fluoropolymer pressure vessels in a CEM MARS5 microwave digestion system. Digested samples were diluted with 2% v/v nitric acid prior to analysis to target an estimated 1–100 µg/L in the analytical solution. Analysis was performed on an Agilent 8900 ICP-MS attached to an Agilent SPS-4 autosampler. The sample introduction system also includes a standard Scott double pass cooled spray chamber operated at 2 °C, a 2.5 mm i.d. Agilent glass torch, and a 200 µL/min Micromist nebulizer. The ICP-MS was fitted with standard nickel cones and operated in no gas mode. Before analysis, all solutions were further diluted by 50% by teeing in an internal standard solution of 2 µg/L platinum and rhodium (High Purity Standards) prepared with 2% v/v nitric acid to correct for instrumental drift. Procedural blanks were run in parallel with samples and averaged 14 ng contributing to less than 2% of the analytical signal and are regarded as negligible. Reproducibility is estimated to be 2.8% RSD from repeated analyses (*n* = 11) over 2 analytical sessions of an in-house QC sample prepared from a polymer of know Pd composition.

### Electrochemical characterization

UV-Vis absorption spectra were recorded on UV-1601 (λmax 1100 nm) UV-VIS Shimadzu UV-Vis spectrometers. Solution UV-Vis absorption spectroscopy was conducted on chloroform solutions with a polymer concentration of 0.006 mg mL^−1^. UV-Vis absorption spectra were also recorded for thin films of the polymers on glass substrates. Cyclic voltammetry was performed employing an Autolab PGSTAT101 with a standard three-electrode configuration, including polymer-coated glassy carbon rod as the working electrode, a platinum wire as the counter electrode and an Ag/AgCl wire electrode as the reference electrode. The polymer was drop cast onto the glassy carbon electrode from 5 mg mL^−1^ solutions and allowed to air dry before each measurement. The supporting electrolyte employed was a 0.1 М tetrabutylammonium hexafluorophosphate in acetonitrile solution or 0.1 M aqueous NaCl solution and was degassed for 30 min before use. The scan rate was 0.1 V s^−1^ and the second and third scans are displayed in each figure herein. Spectroelectrochemical measurements were obtained from thin films of the polymer on ITO glass slides with the corresponding semiconductor. This was then connected in the same three-electrode configuration as for the cyclic voltammetry experiment and placed within a cuvette holder. The resulting electrochemical setup was then configured to reside inside the UV-1800 Shimadzu UV-Vis spectrometer previously employed for determining the optical properties of the polymers.

### Transistor fabrication and characterization

OECT substrates were fabricated following previously reported photolithographic techniques^46^. The OECT devices featured gold contacts making up a channel with width = 100 µm and length = 10 µm. To mitigate the problems with the parylene lift-off with p-type polymers, the substrates underwent a pre-deposition surface treatment to promote adhesion of the polymer film to the channels and electrodes^66^. The polymer solutions (5 mg mL^−1^) were deposited by spin-coating at 1000 rpm and 500 rpm for the p- and n-type polymers, respectively, for 45 s before air drying and peeling off the sacrificial parylene layer. Transistor properties were measured using an aqueous 0.1 М NaCl solution as the supporting electrolyte and an Ag/AgCl pellet electrode as the gate electrode and presented data is an average across 6 devices. Output and transfer characteristics, as well as stability measurements were recorded by connecting OECTs to an IviumStat potentiostat. Data was recorded through custom built LabVIEW software. Electrochemical impedance spectroscopy was performed using the same potentiostat, employing 600 µm × 600 µm polymer coated gold electrodes as the working electrode, with an Ag/AgCl pellet electrode as the gate electrode. For electrochemical impedance spectroscopy experiments a frequency range between 100 kHz to 1 Hz and an AC amplitude of 10 mV over a DC voltage above the threshold for the polymer. All OFET films were prepared and characterized under a nitrogen atmosphere. Borosilicate substrates were cleaned by sequential ultrasonication in a dilute Extran 300 detergent solution and deionized water for 30 min each. Then the substrates were cleaned with acetone and isopropyl alcohol by ultrasonication for 10 min each. As source-drain electrodes, 35 nm of Au with 5 nm of Al as the sticking layer was thermally evaporated at 2 × 10^−6^ mbar. The substrates with patterned Au/Al source–drain contacts were subjected to a UV-ozone treatment step for 20 min. The channel width and length of the transistors were 1000 μm/40 μm. Before the film deposition, the polymers were dissolved in anhydrous chloroform. The solutions were kept under stirring on a hot plate at room temperature for overnight. As gate dielectric, the mixture of Cytop (CTL-809M) and its solvent (CT-SOLV 180) with a volume ratio of 2:1 was spin-coated at 1200 rpm for 60 s. The Cytop film was then thermally annealed at 50 °C for 2 h before the deposition of Al as a gate electrode. The electrical characteristics of the OTFTs were monitored by a probe station placed in an N_2_-filled glove box that is connected to an Agilent B1500A semiconductor parameter analyzer. The saturation-regime mobility (*μ*_sat_) of the transistor was determined using the equation ${\mu }_{{{{{{{\rm{sat}}}}}}}}=2L/W{C}_{i}{(\frac{{{{{{\rm{d}}}}}}{{I}_{{{{{{{\rm{d}}}}}}}}}^{1/2}}{{{{{{\rm{d}}}}}}{V}_{{{{{{\rm{g}}}}}}}})}^{2}$, where *I*_d_ is the source–drain current, *C*_*i*_ is the capacitance per unit area, *L* is the channel length, *W* is the channel width, and *V*_g_ is the gate voltage.

## Supplementary information

Supplementary Information

## Data Availability

The authors declare that the data supporting the findings of this study are available within the paper and its Supplementary Information files. Additional data are available from the corresponding author upon request. The data acquired at the University of Bern that support the results of this study are available as open access in BORIS at 10.48620/183.
