# Supplementary material for: The effect of residual palladium on the performance of organic electrochemical transistors
Source: Nat Commun. 2022 Dec 27;13:7964. doi: 10.1038/s41467-022-35573-y (PMC9794802; doi:10.1038/s41467-022-35573-y)
Supplement: Supplementary file 1 — Supplementary Information [file 41467_2022_35573_MOESM1_ESM.pdf]

SUPPLEMENTARY INFORMATION

The effect of residual palladium on the performance of organic electrochemical transistors

Griggs et al.

SUPPLEMENTARY FIGURES

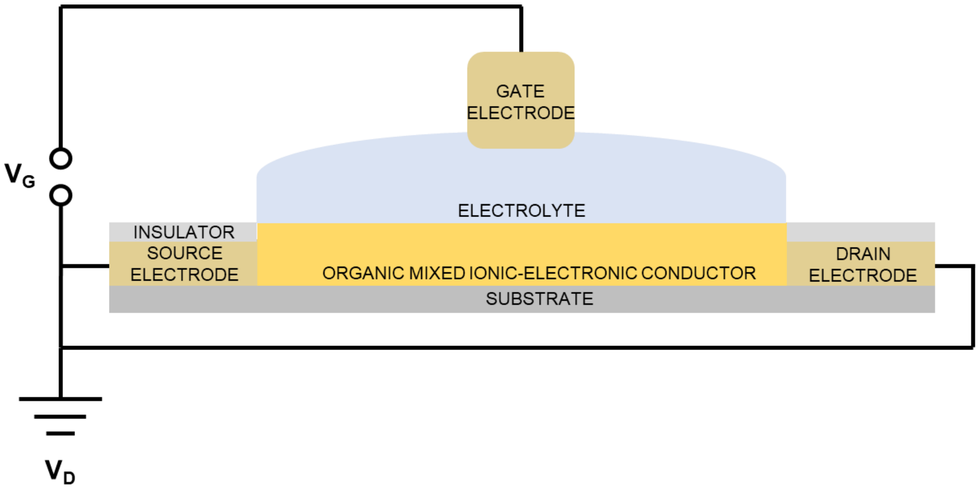

Supplementary Figure 1. Schematic architecture of an OECT, where V_D is the applied source-drain bias and V_G is the gate voltage.

Monomer synthesis routes

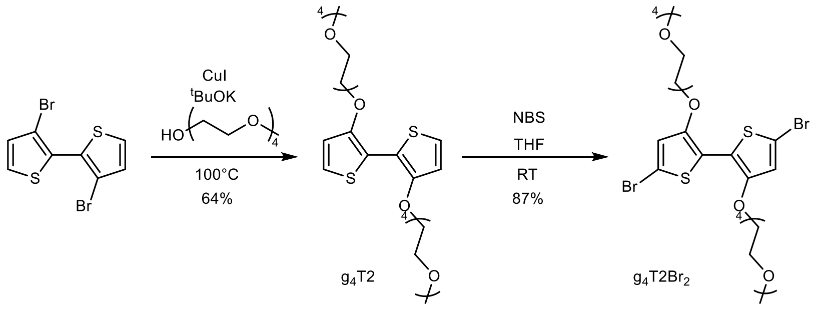

Supplementary Figure 2. Synthesis of g_4T2-Br_2.

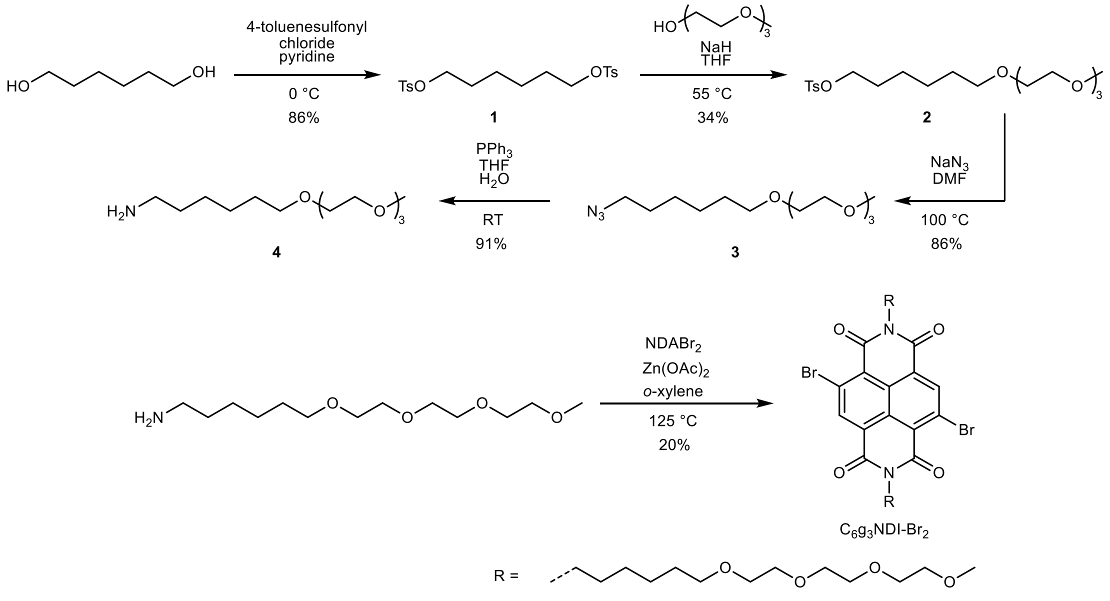

Supplementary Figure 3. Synthesis of C_6g_3NDI-Br_2.

Polymer syntheses

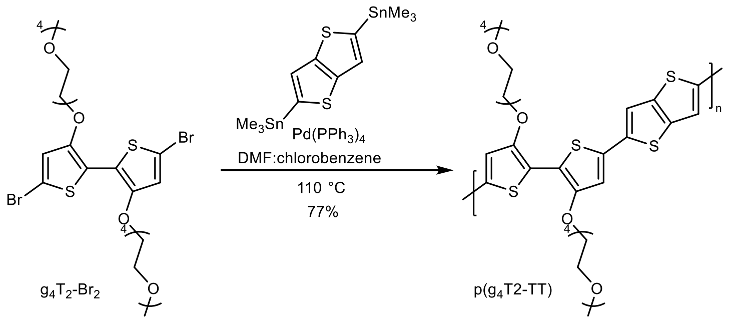

Supplementary Figure 4. Synthesis of p(g_4T2-TT).

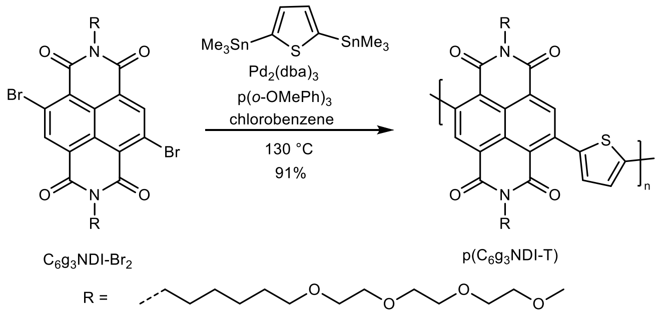

Supplementary Figure 5. Synthesis of p(C_6g_3NDI-T).

Monomer NMR of $g_4T2\text{-}Br_2$

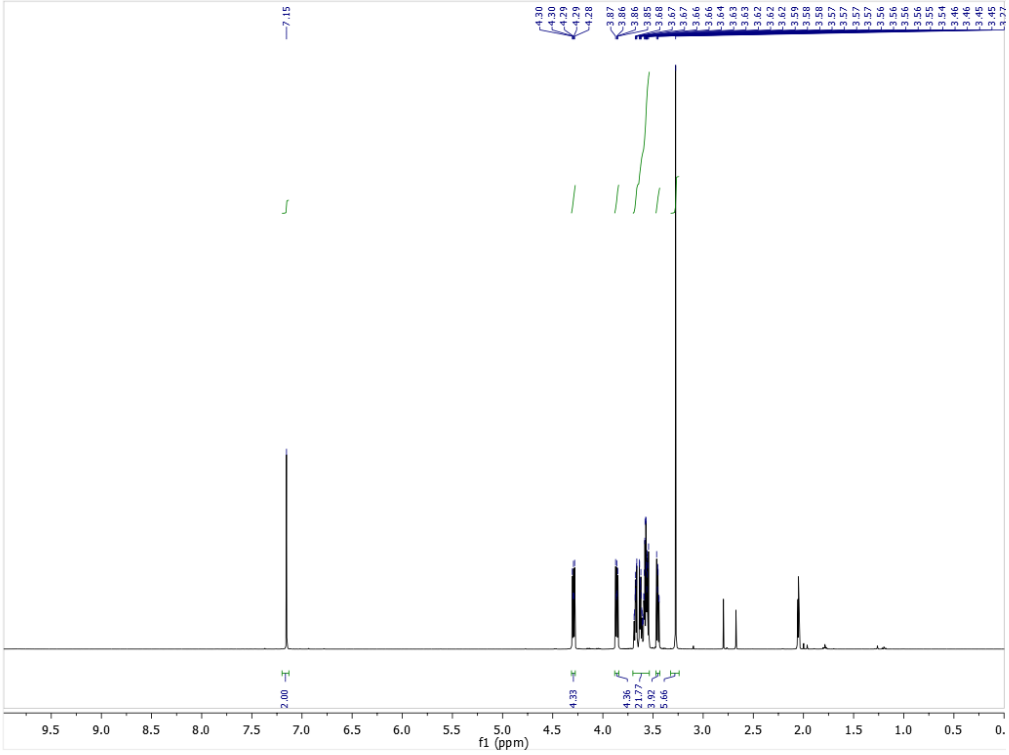

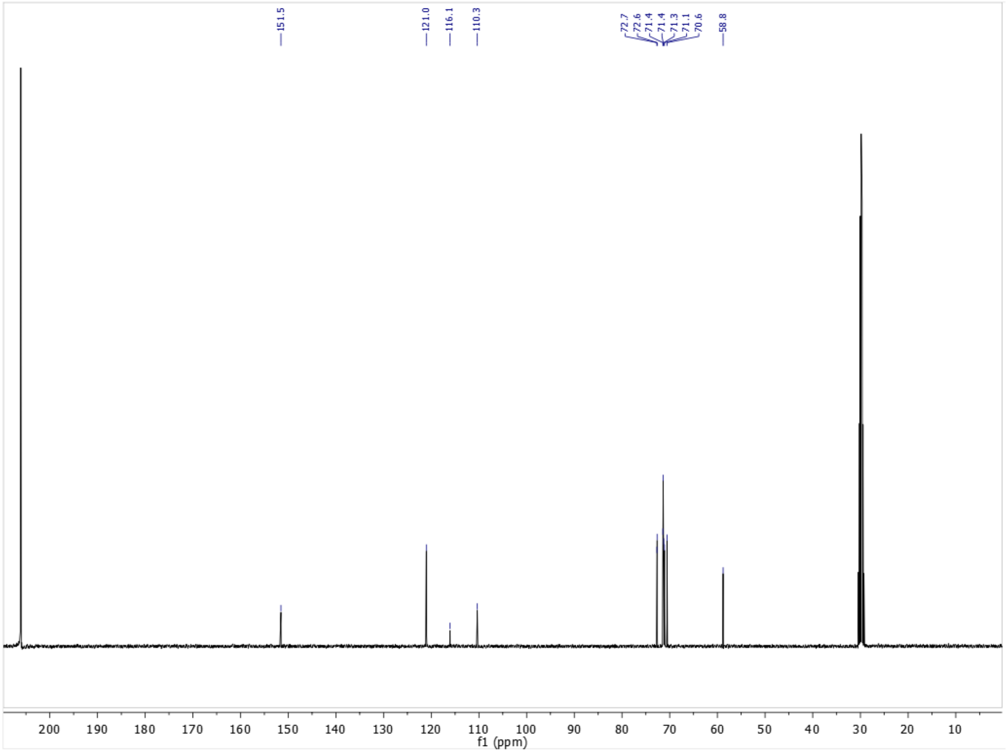

Supplementary Figure 6. ^{1}H NMR (400 MHz) and ^{13}C NMR (101 MHz) spectra of (g_4T2Br_2) in acetone-d_6.

Monomer NMR of $C_6g_3NDI\text{-}Br_2$

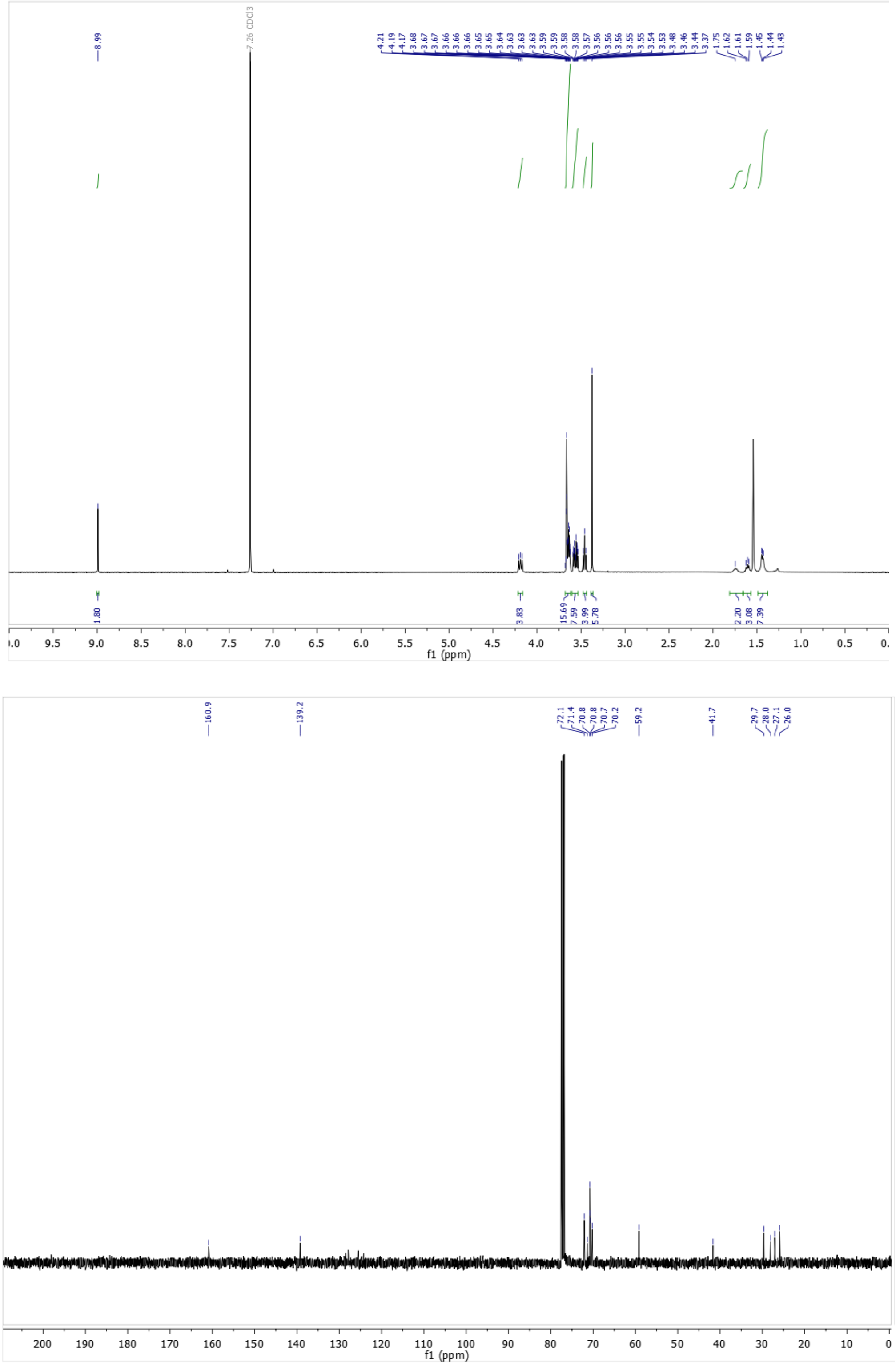

Supplementary Figure 7. ^{1}H NMR (400 MHz) and ^{13}C NMR (101 MHz) spectra of ($C_6g_3NDI\text{-}Br_2$) in $CDCl_3$.

Polymer NMR of p(g_4T2-TT)

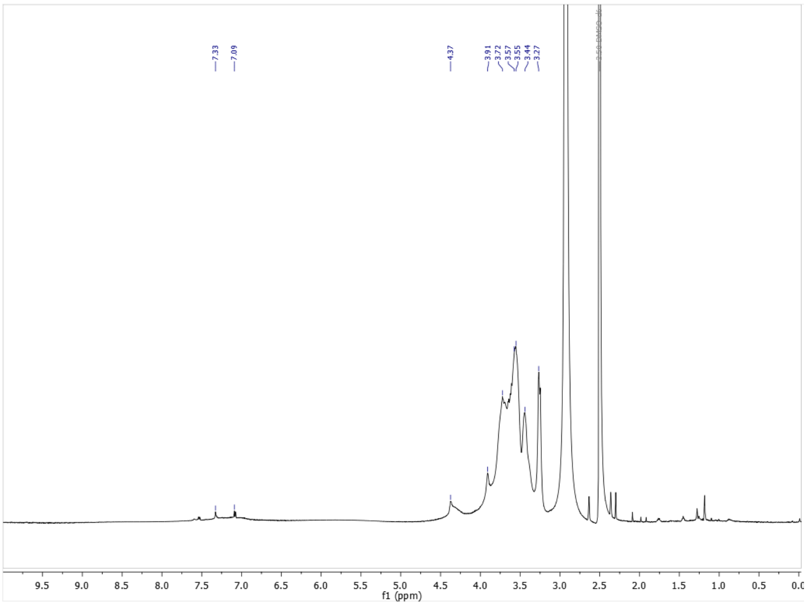

Supplementary Figure 8. ^{1}H NMR (400 MHz) spectra of p(g_4T2-TT) in DMSO-d_6.

Polymer NMR of p(C_6g_3NDI-T)

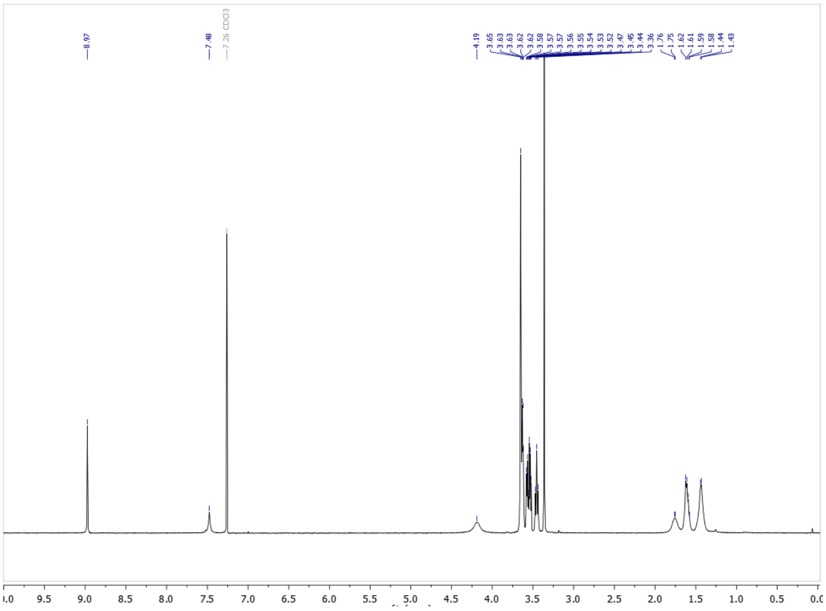

Supplementary Figure 9. ^{1}H NMR (400 MHz) spectra of p(C_6g_3NDI-T) in $CDCl_3$.

GPC measurements

(a)

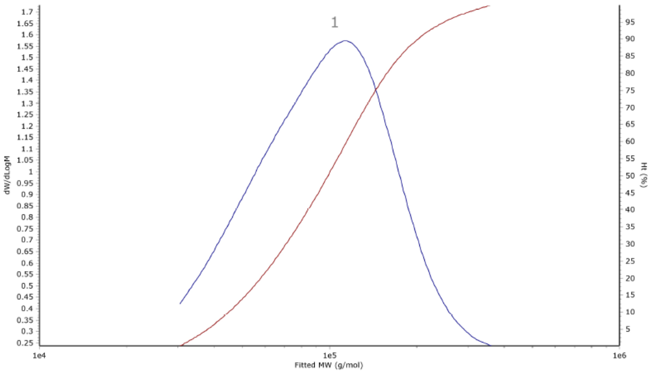

(b)

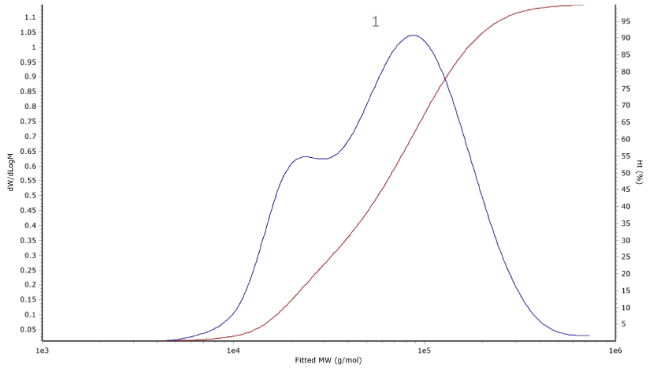

Supplementary Figure 10. Gel permeation chromatography traces for (a) p(g_4T2-TT) and (b) p(C_6g_3NDI-T) unpurified in DMF and chloroform respectively at 40 °C.

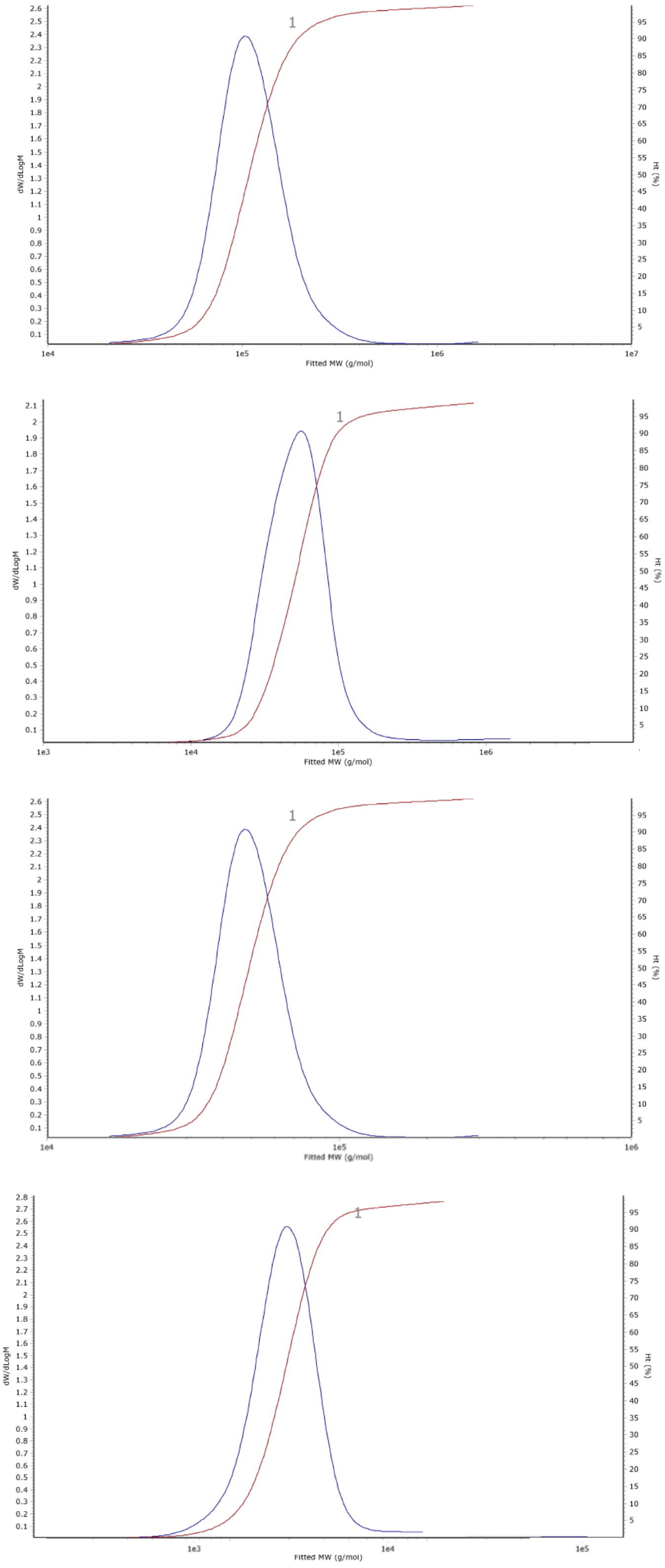

Supplementary Figure 11. Gel permeation chromatography traces for fraction 1-4 of p(g_4T2-TT) in DMF at 40 °C.

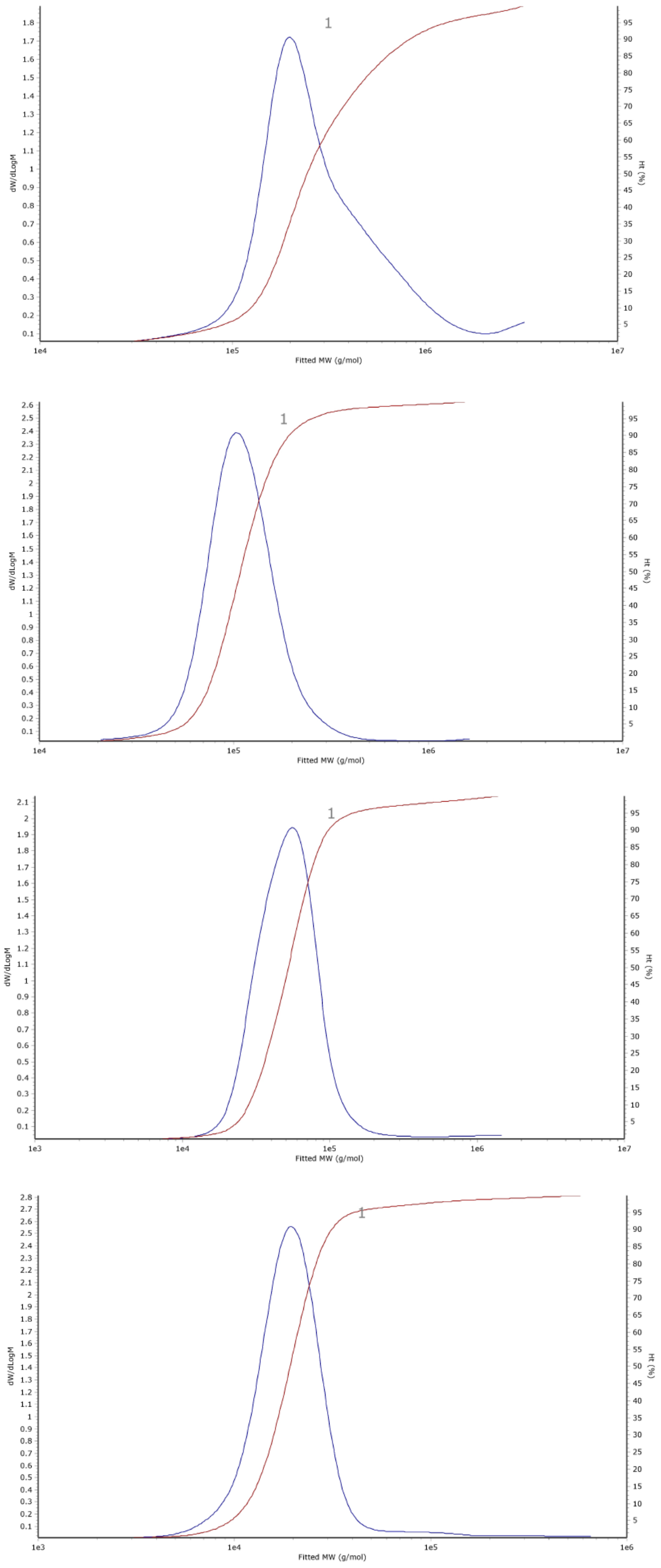

Supplementary Figure 12. Gel permeation chromatography traces for fractions 1-4 of p(C_6g_3NDI-T) in chloroform at 40 °C.

Organic CV measurements

(a)

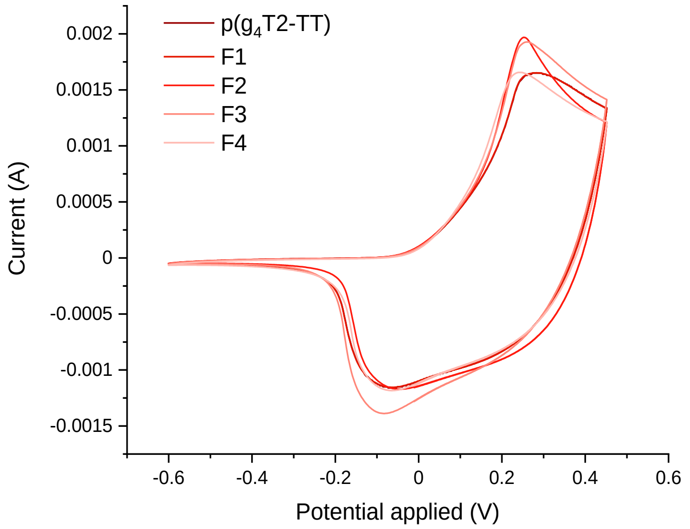

(b)

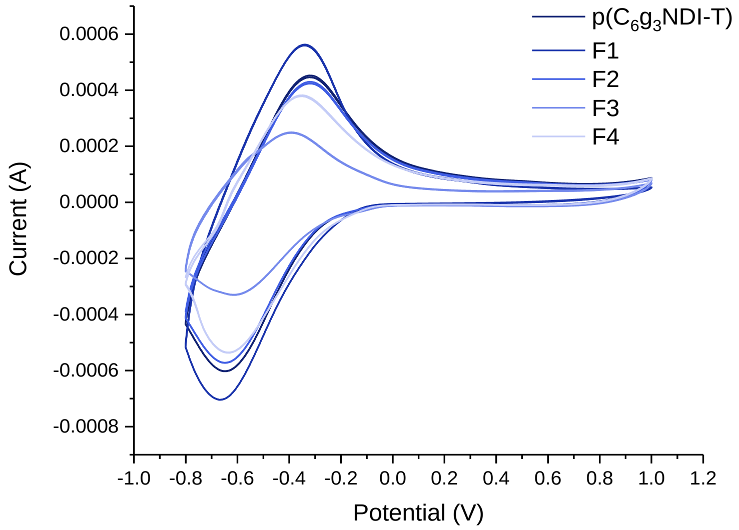

Supplementary Figure 13. Cyclic voltammograms of (a) p(g_4T2-TT) and (b) p(C_6g_3NDI-T) unpurified (darkest blue) and F1-F4 (lightest blue). Determined by cyclic voltammetry of the polymer thin films on glassy carbon working electrode vs Ag/AgCl in acetonitrile with 0.1 M tetrabutylammonium hexafluorophosphate as the supporting electrolyte, using a platinum counter electrode.

UV-Vis absorption spectroscopy

(a)

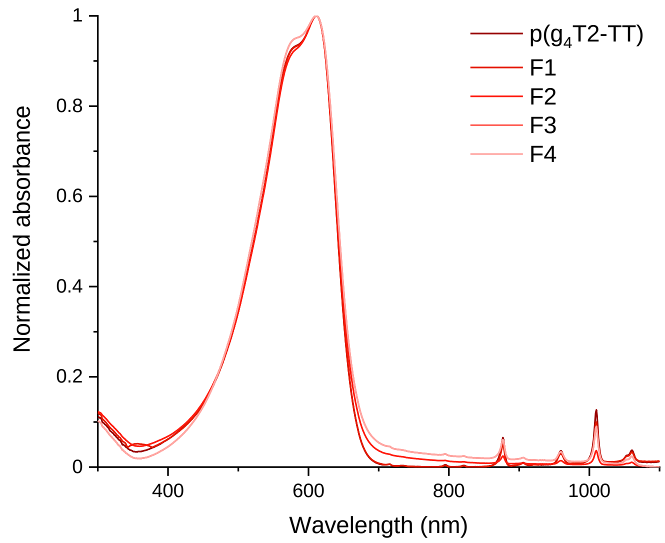

(b)

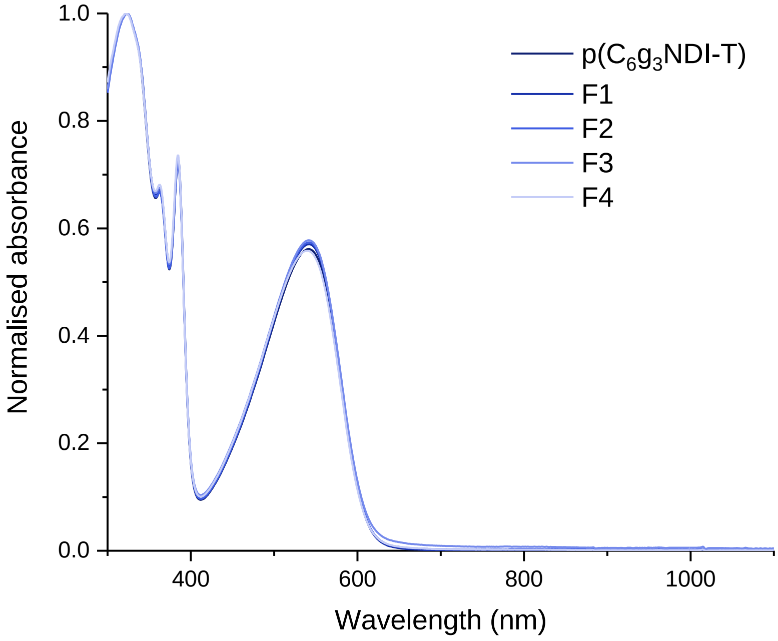

Supplementary Figure 14. Normalized UV-Vis absorption spectra in chloroform of (a) p(g_4T2-TT) and (b) p(C_6g_3NDI-T) unpurified and F1-F4.

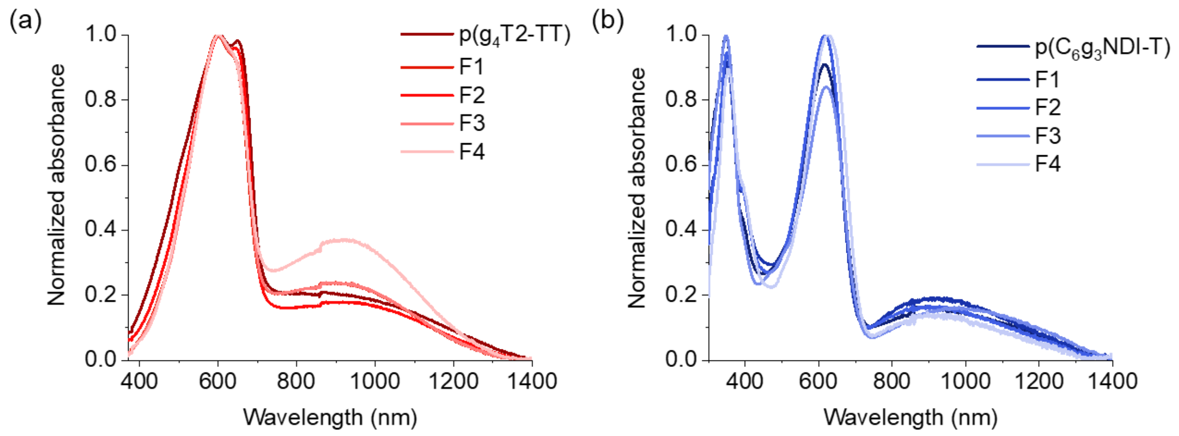

Supplementary Figure 15. Normalized UV-Vis-NIR absorption spectra of thin films on glass substrates of (a) p(g_4T2-TT), with average thickness of three films of 83 nm, 99 nm, 95 nm and 57 nm for the unpurified batch and F2-4, respectively (Figure S15 (c)), and (b) p(C_6g_3NDI-T), with average thickness of three films of 57 nm, 53 nm, 53 nm, 50 nm and 41 nm for the unpurified batch and F1-4, respectively Figure S15 (d)).

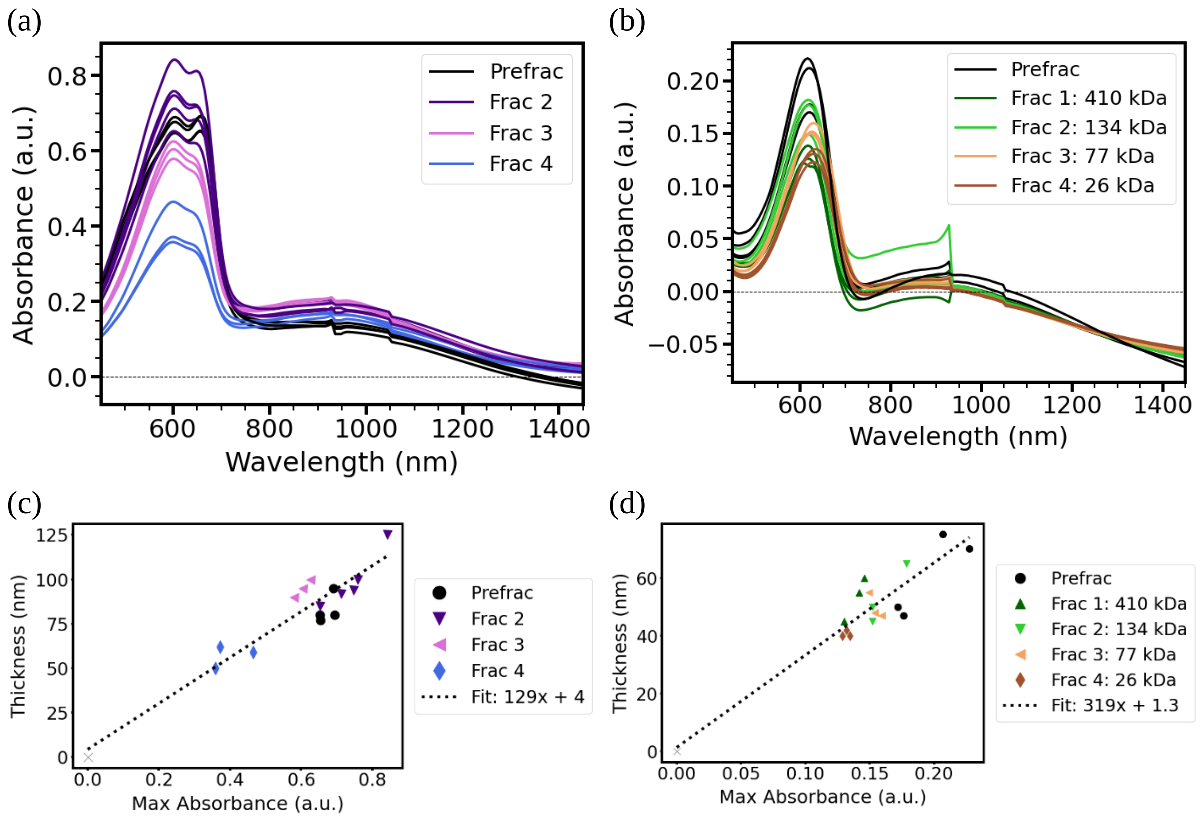

Supplementary Figure 16. Absolute UV-Vis absorption spectra of thin films on glass substrates of (a) p(g_4T2-TT) and (b) p(C_6g_3NDI-T) unpurified and F1-F4, and the thickness of each of the films of (c) p(g_4T2-TT) and (d) p(C_6g_3NDI-T) unpurified and F1-F4. This supports the molecular weight determination that F1 is the heaviest and F4 is the lightest because as molecular weight decreases, the absorbance decreases as the films become thinner, suggesting a lower viscosity for the same 5 mg mL^{-1} concentration.

GIWAXS

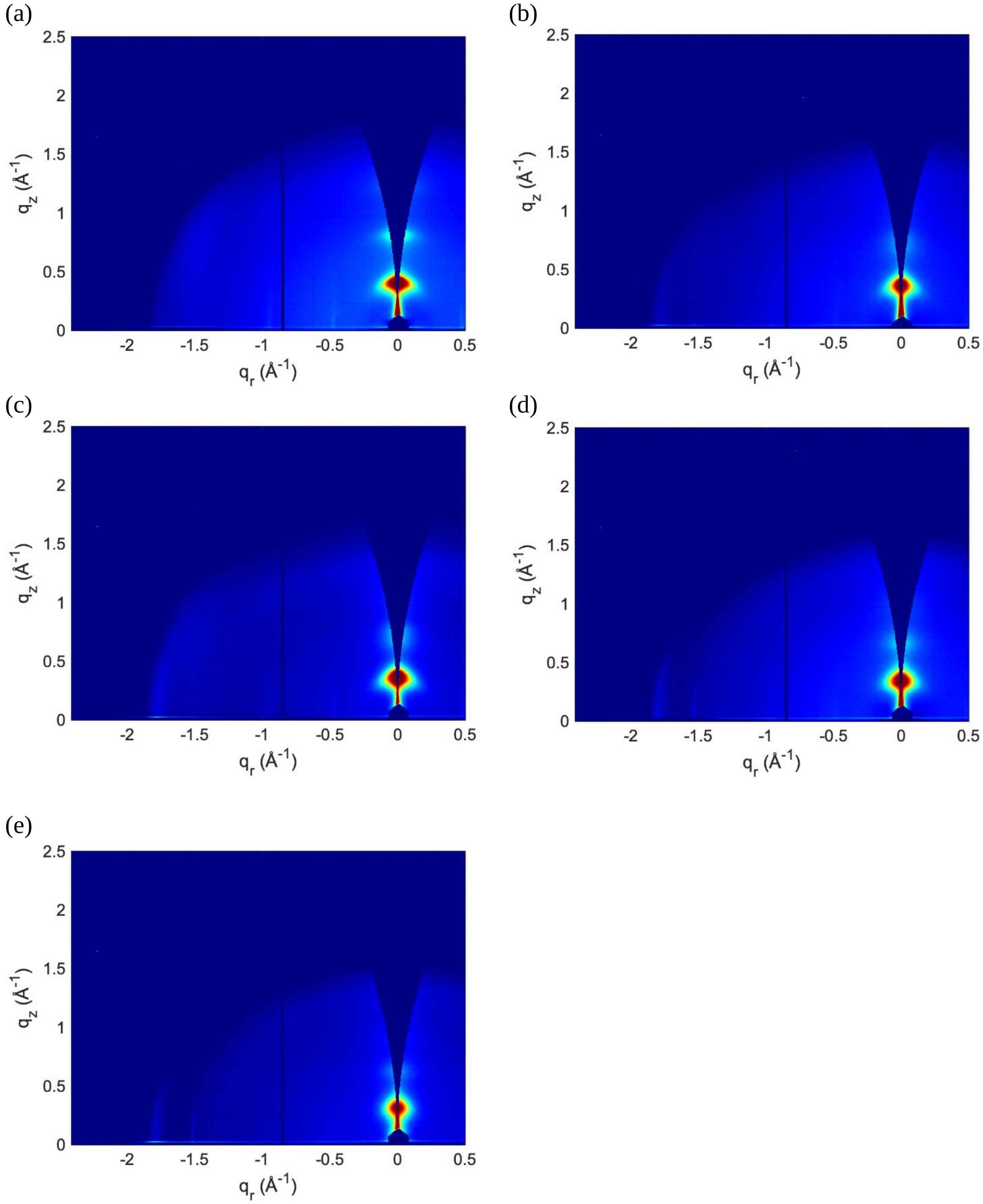

Supplementary Figure 17. Ex situ GIWAXS q_r-q_z plots of neat (as cast) p(g_4T2-TT) (a) unpurified and (b)-(e) fractions 1-4, respectively.

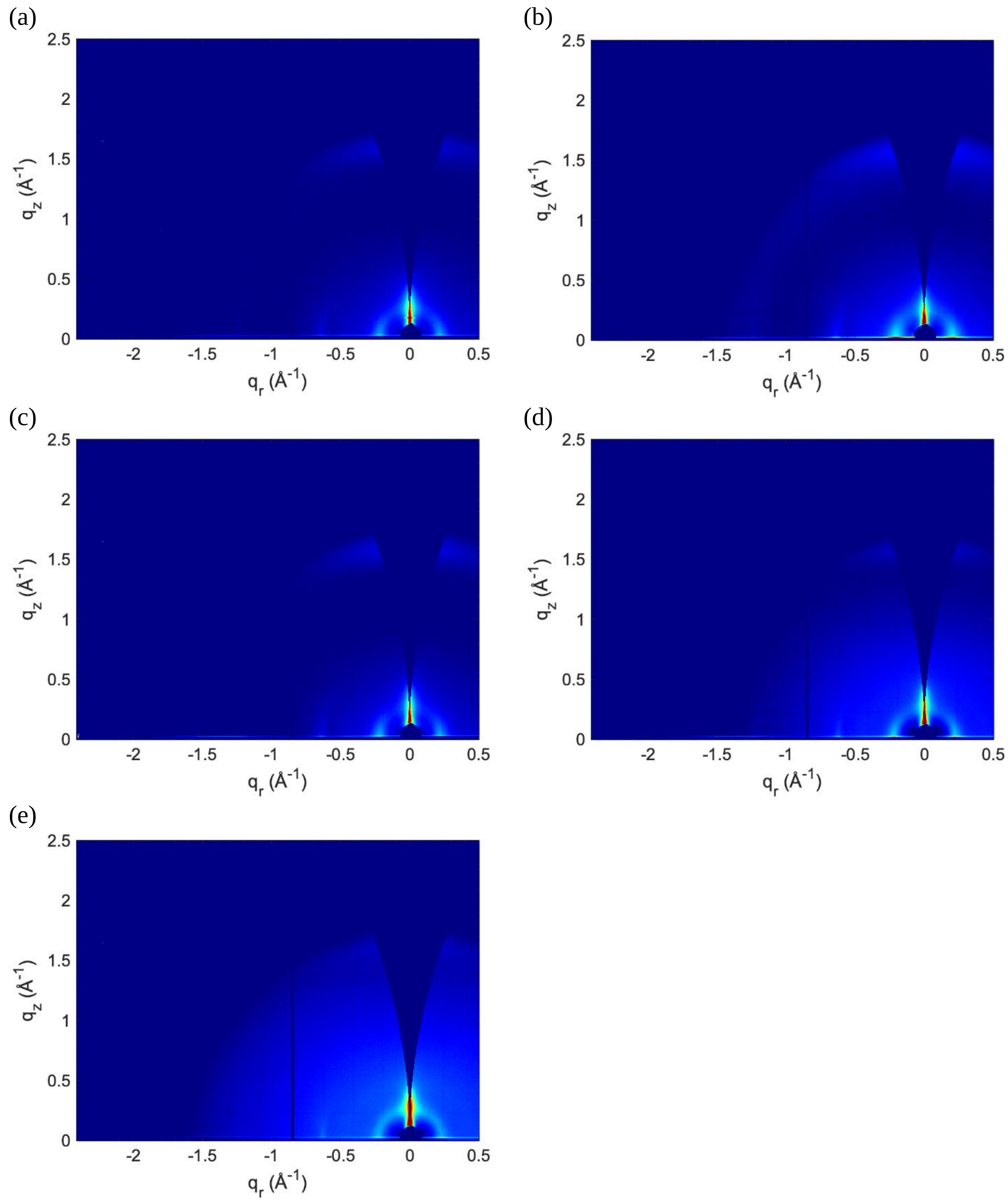

Supplementary Figure 18. Ex situ GIWAXS q_r-q_z plots of neat (as cast) p(C_6g_3NDI-T) (a) unpurified and (b)-(e) fractions 1-4, respectively.

Aqueous CV measurements

(a)

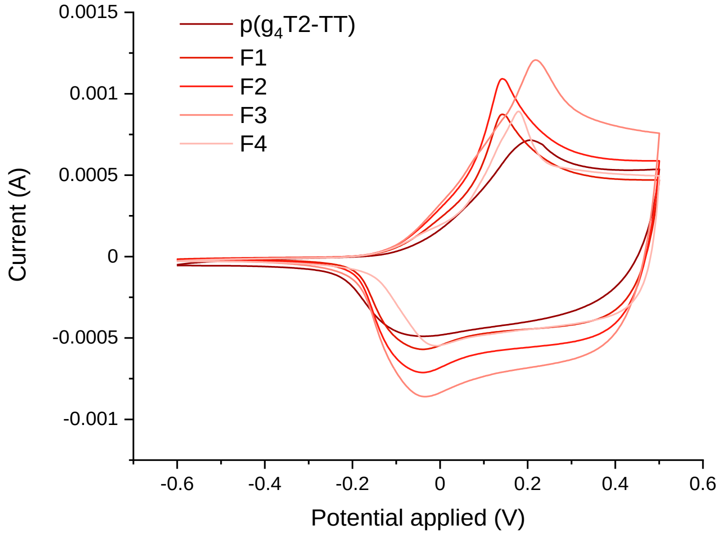

(b)

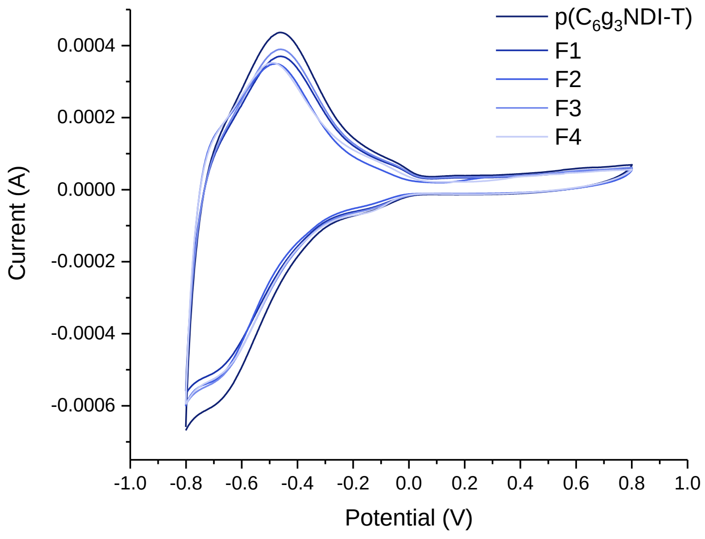

Supplementary Figure 19. Cyclic voltammograms of (a) p(g_4T2-TT) and (b) p(C_6g_3NDI-T) unpurified (darkest blue) and F1-F4 (lightest blue). Determined by cyclic voltammetry of the polymer thin films on glassy carbon working electrode vs Ag/AgCl in 0.1 M aqueous NaCl as the supporting electrolyte, using a platinum counter electrode.

Spectroelectrochemical measurements

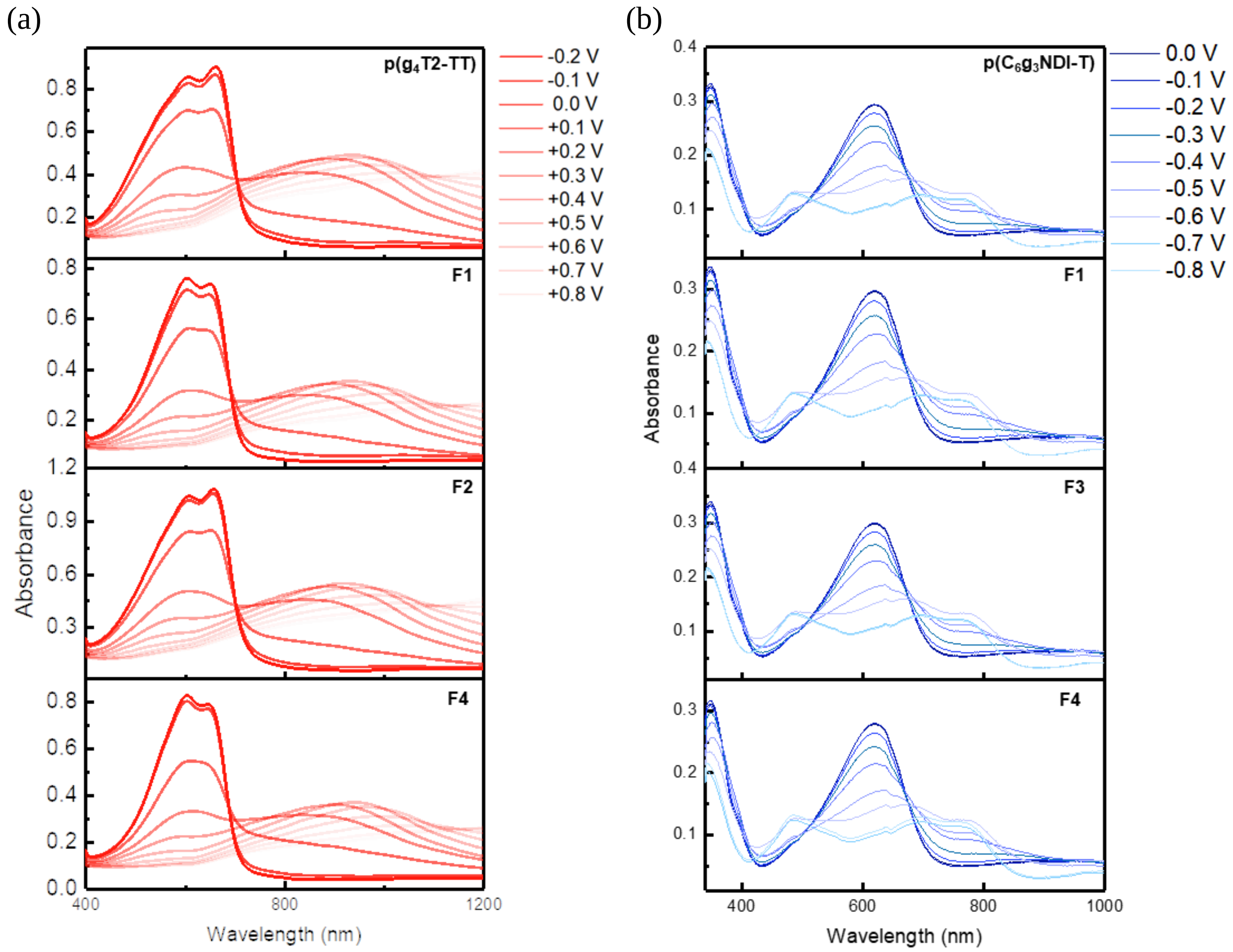

Supplementary Figure 20. Evolution of the UV–vis absorption spectra of (a) p(g_4T2-TT) unpurified and fractions 1, 2 and 4 during charging from -0.2 V (dark red) to +0.8 V (light pink) versus Ag/AgCl in a 0.1 M NaCl aqueous solution and (b) p(C_6g_3NDI-T) unpurified and fractions 1, 3 and 4 during charging from 0 V (dark blue) and -0.8 V (light blue) versus Ag/AgCl in a 0.1 M NaCl aqueous solution.

(a)

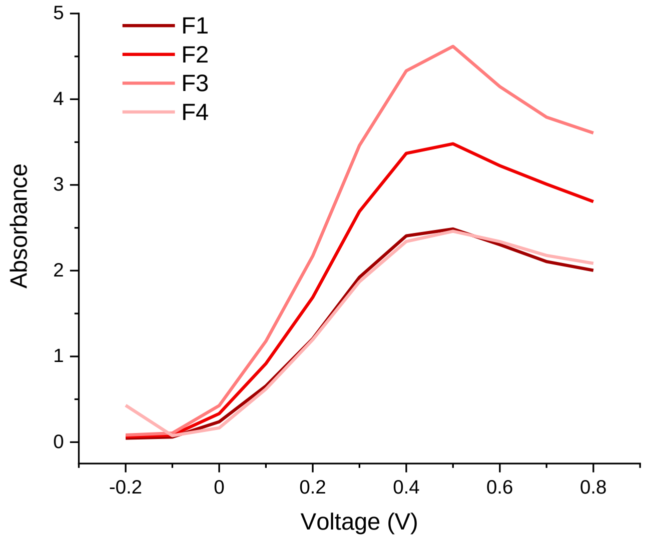

(b)

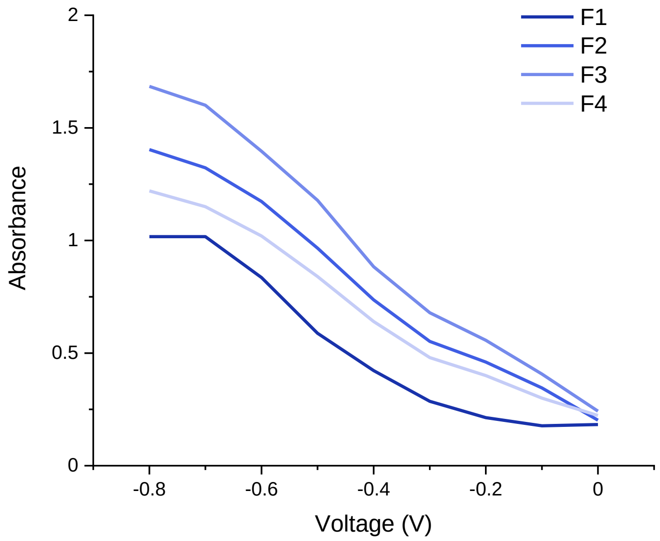

Supplementary Figure 21. (a) The absorbance at 1000 nm at each voltage, when the π-π* band (at 605 nm) is normalised to one, for p(g_4T2-TT) and (b) the absorbance at 790 nm at each voltage, when the π-π* band (at 617 nm) is normalised to one, for p(C_6g_3NDI-T). These graphs support the trends observed in *C**.

OECT measurements

(a)

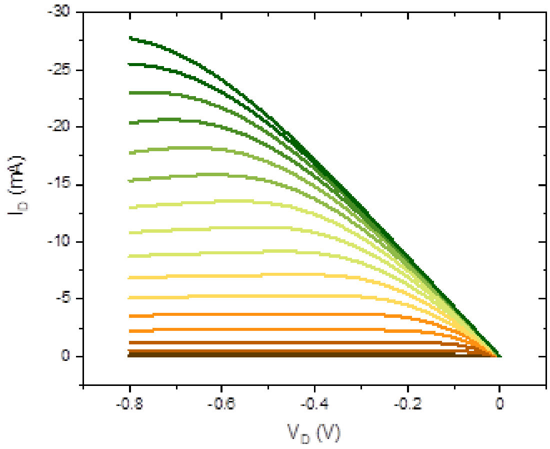

(b)

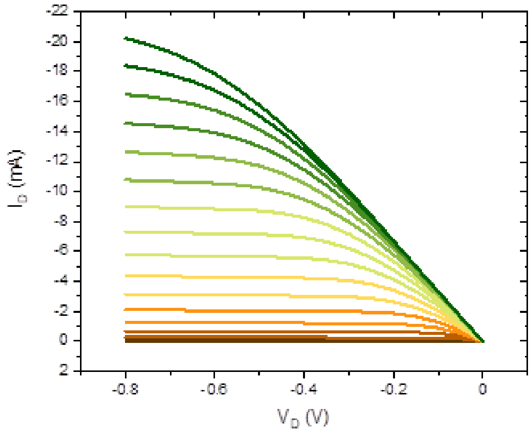

(c)

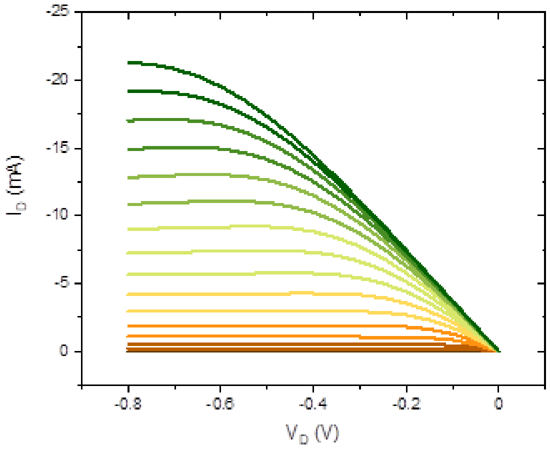

(d)

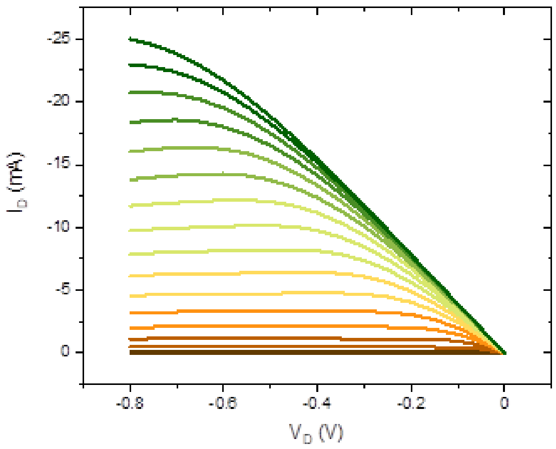

(e)

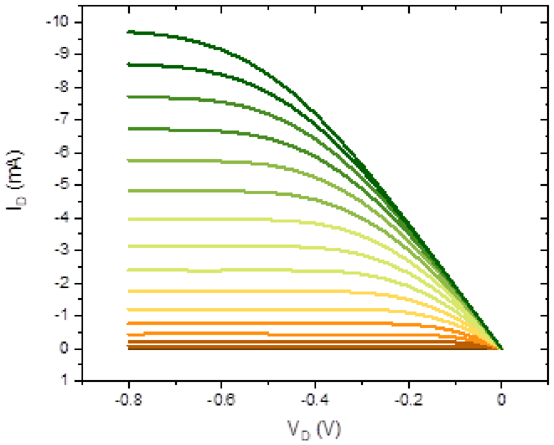

Supplementary Figure 22. Performance of the OECTs (W = 100 µm, L = 10 µm, sweep rate of 0.1 V s^{-1}) in an aqueous 0.1 M NaCl solution in ambient conditions showing the output characteristics (V_G = 0.0 to 0.8 V, ΔV_G = 0.05 V) for p(g$_4$T2-TT) (a) unpurified and (b)-(e) fractions 1-4, respectively.

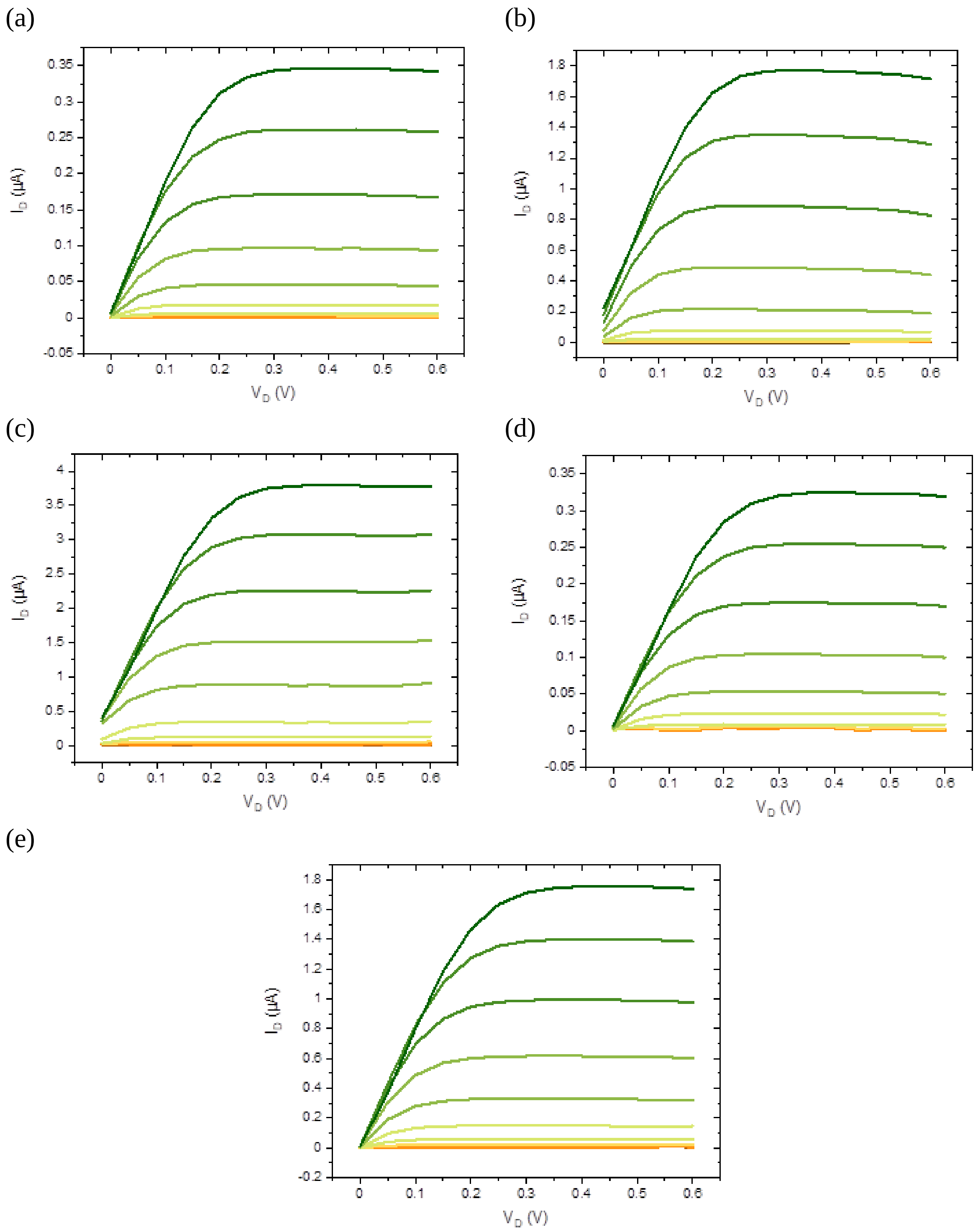

Supplementary Figure 23. Performance of the OECTs (W = 100 μm, L = 10 μm, sweep rate of 0.1 V s^{-1}) in an aqueous 0.1 M NaCl solution in ambient conditions showing the output characteristics (V_G = 0.0 to 0.6 V, ΔV_G = 0.05 V) for p(C_6g_3NDI-T) (a) unpurified and (b)-(e) fractions 1-4, respectively.

(a)

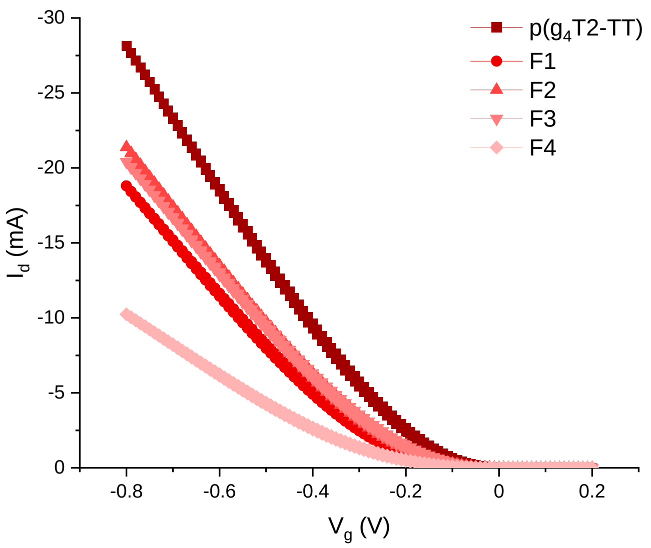

(b)

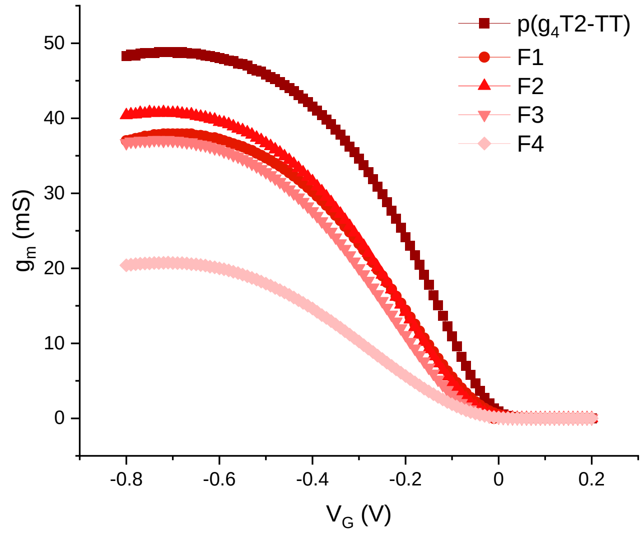

Supplementary Figure 24. (a) Transfer curves and (b) transconductance versus gate voltage for p(g_4T2-TT). OECT performance measured in devices where *W* = 100 µm and *L* = 10 µm, at a sweep rate of 0.1 V s^{-1} in 0.1 M $NaCl_{(aq)}$ operated in a diode-like configuration with the gate and drain shorted together in ambient conditions.

(a)

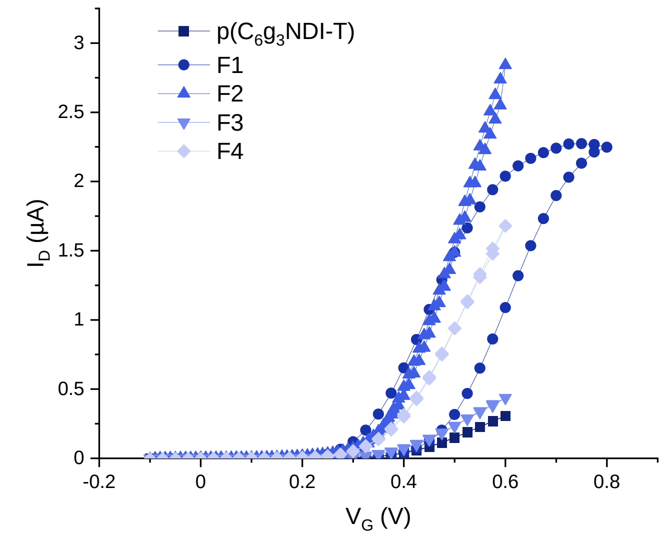

(b)

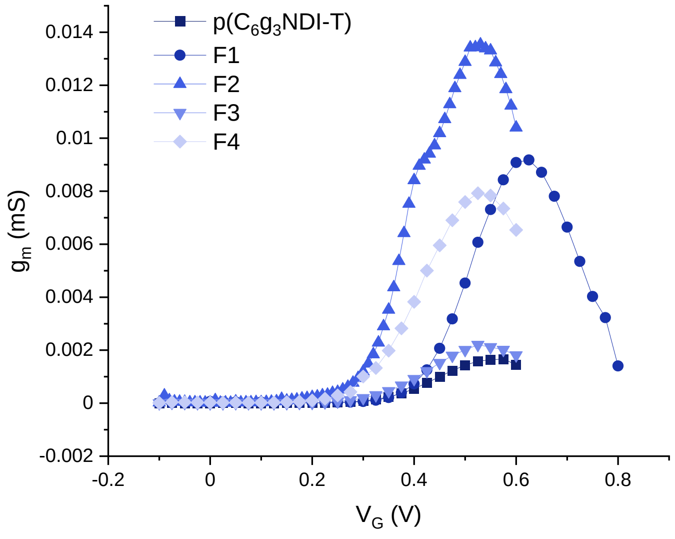

Supplementary Figure 25. (a) Transfer curves and (b) transconductance versus gate voltage for p(C_6g_3NDI-T). OECT performance measured in devices where W = 100 μm and L = 10 μm, at a sweep rate of 0.1 V s^{-1} in 0.1 M $NaCl_{(aq)}$ operated in a diode-like configuration with the gate and drain shorted together in ambient conditions.

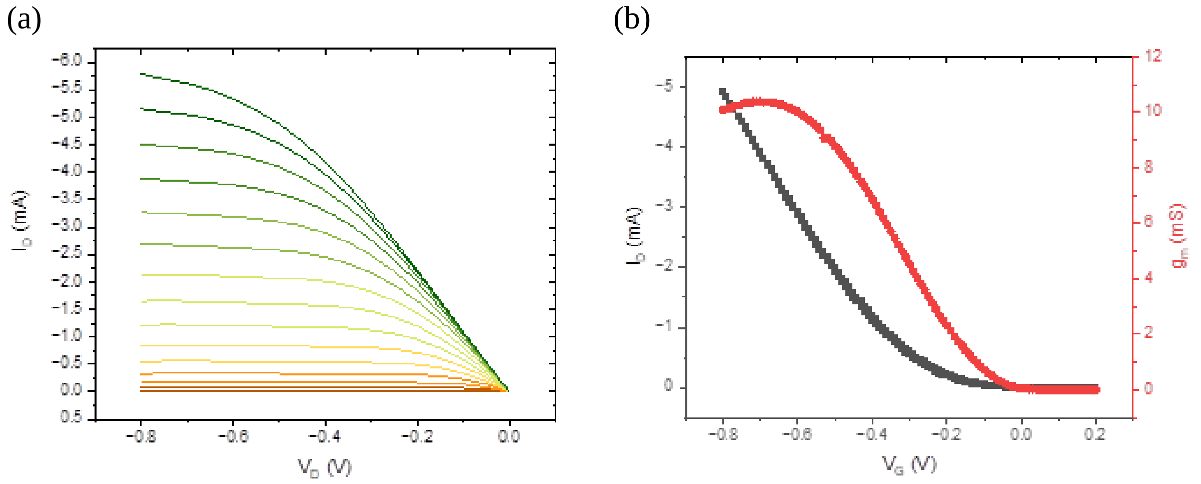

Supplementary Figure 26. (a) Output and (b) transfer and transconductance characteristics for the recombined “mixed” batch of p(g_4T2-TT).

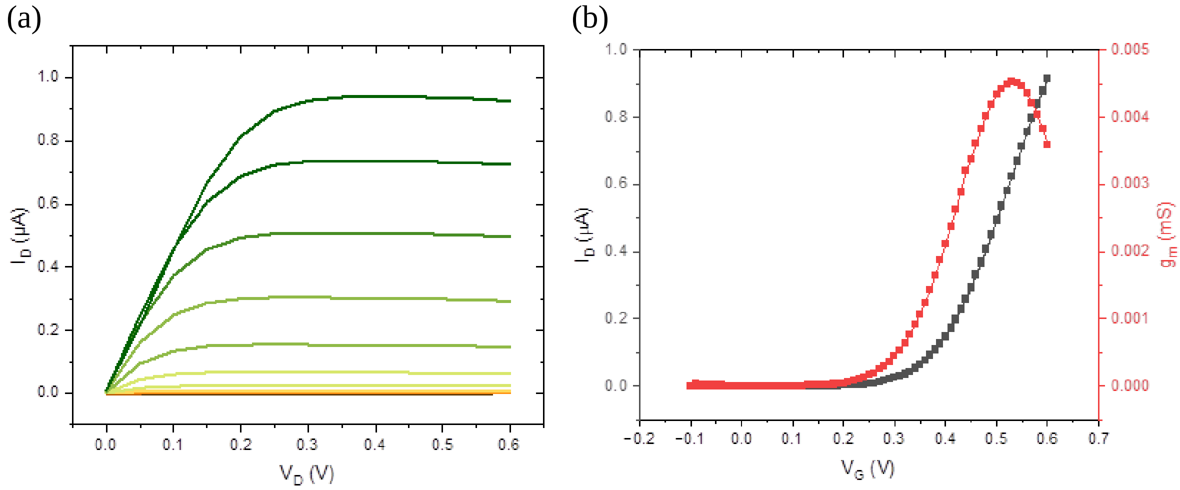

Supplementary Figure 27. (a) Output and (b) transfer and transconductance characteristics for the recombined “mixed” of p(C_6g_3NDI-T).

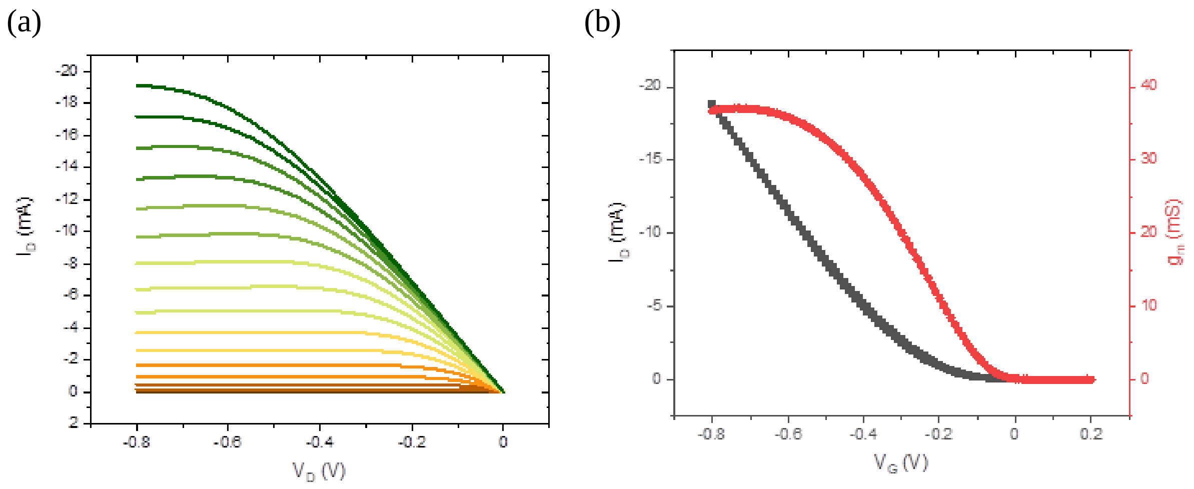

Supplementary Figure 28. (a) Output and (b) transfer and transconductance characteristics for F3 of p(g_4T2-TT) doped with 2000 ppm of Pd.

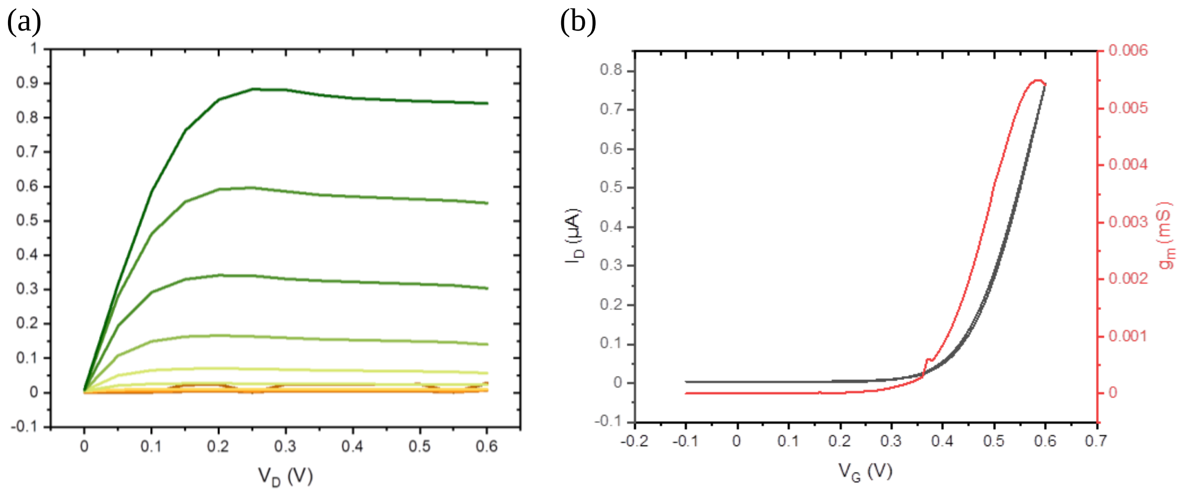

Supplementary Figure 29. (a) Output and (b) transfer and transconductance characteristics for F2 of p(C_6g_3NDI-T) doped with 2000 ppm of Pd.

OFET performance

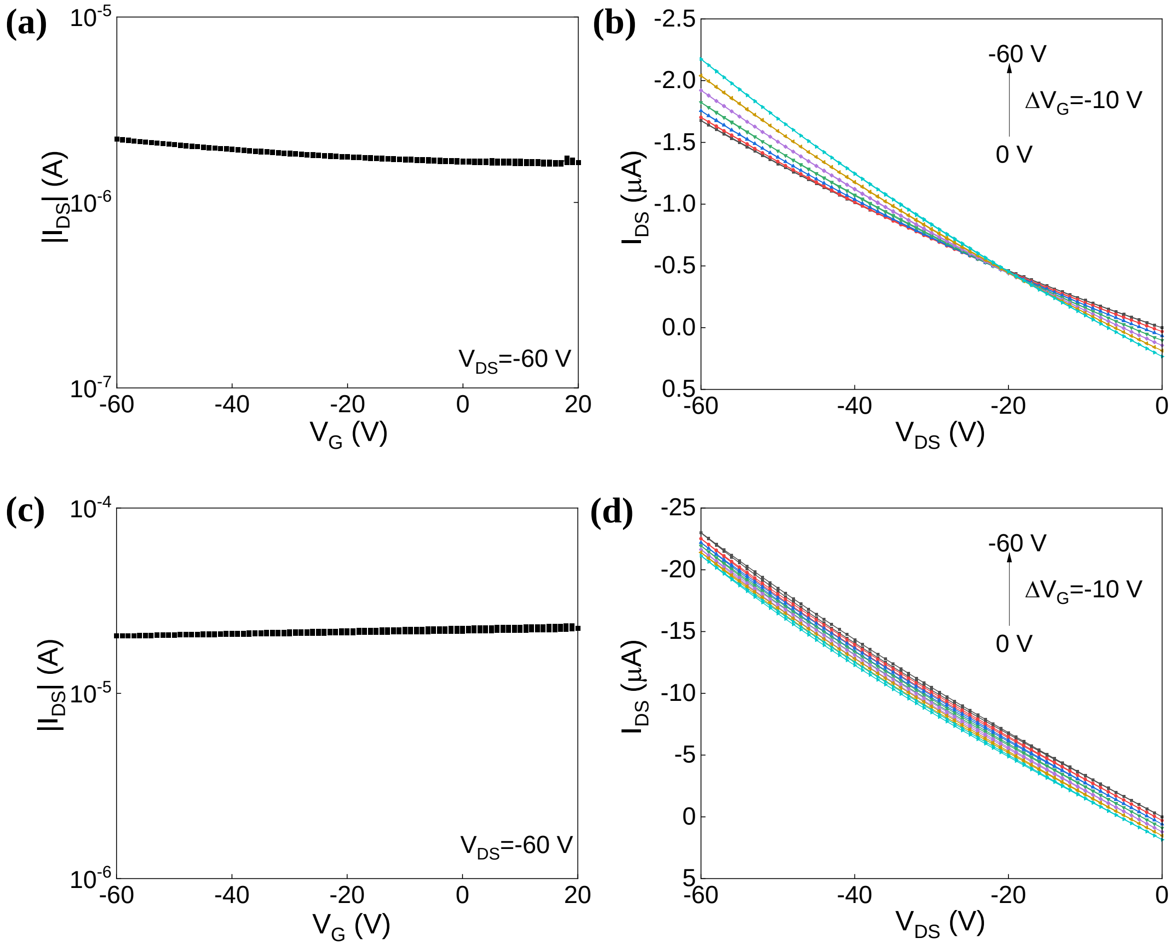

Supplementary Figure 30. (a) The transfer curve and (b) output curve for unpurified p(g_4T2-TT) and (c) the transfer curve and (d) output curve for F3 of p(g_4T2-TT). The output curves are measured for V_G = 0 V to V_G = -60 V in 10 V steps and display conducting properties.

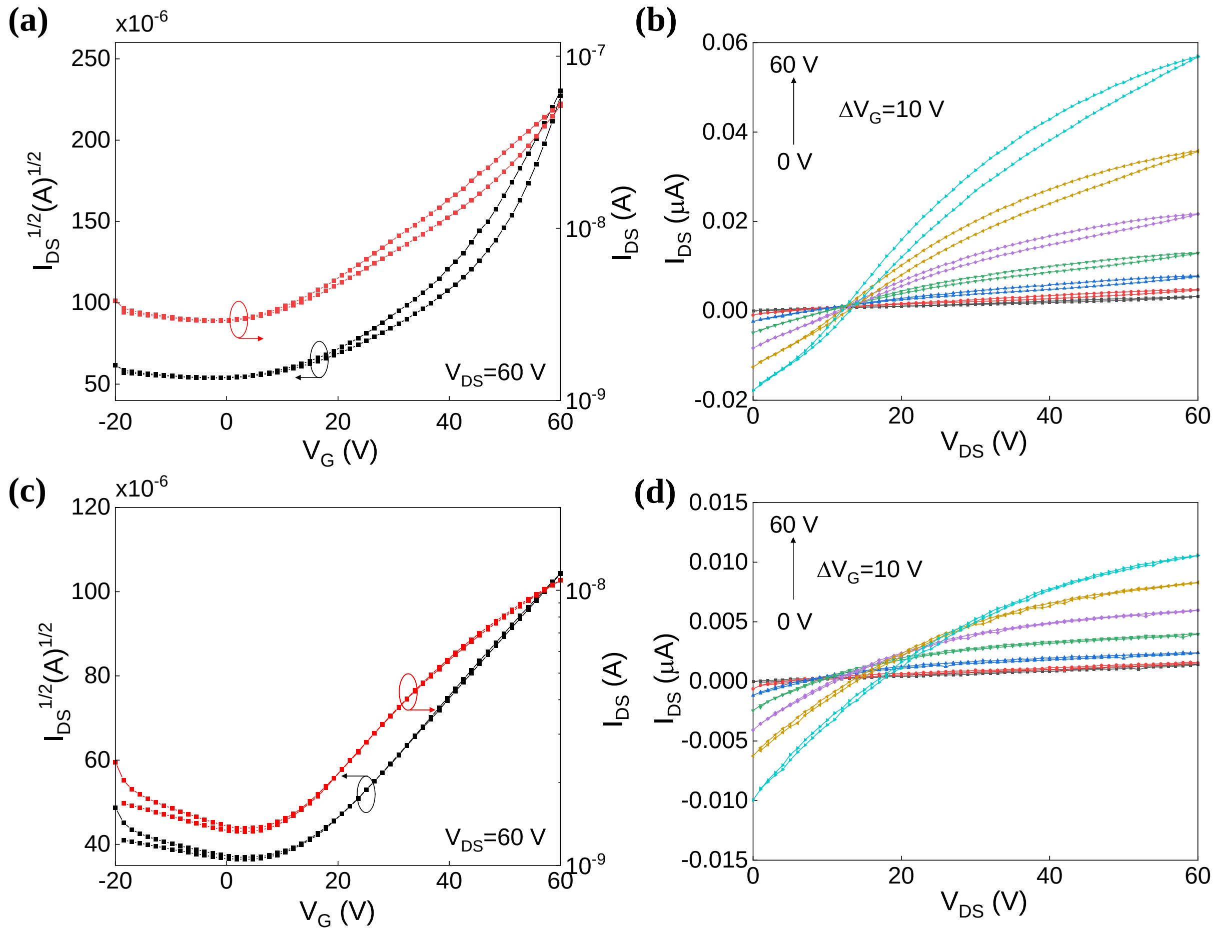

Supplementary Figure 31. (a) The transfer curve and (b) output curve for unpurified p(C_6g_3NDI-T) and (c) the transfer curve and (d) output curve for F2 of p(C_6g_3NDI-T). The transfer curves are recorded in the saturation regime, where V_{DS} = 60 V (black) and the mobility calculated from the linear fit of the drain current square root (red). The output curves are measured for V_G = 0 V to V_G = 60 V in 10 V steps. The non-ideal output characteristics are attributed to the very low currents recorded.

Electrical impedance spectroscopy (EIS)

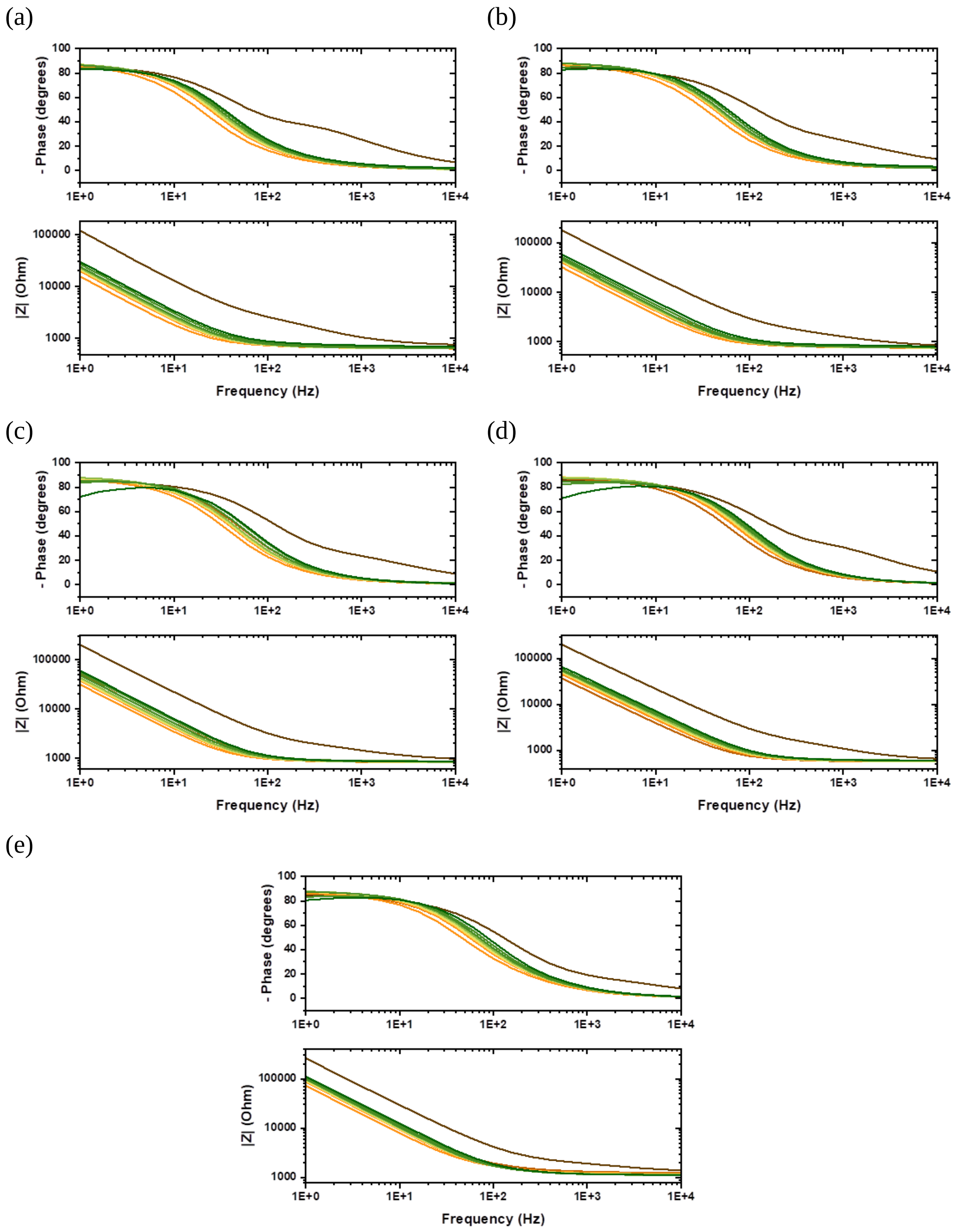

Supplementary Figure 32. Electrochemical impedance spectroscopy of (a) unpurified and (b)-(e) fractions 1-4 of p(g_4T2-TT) showing the impedance magnitude and the phase of the impedance at V_G = -0.8 to 0 V. *C** was calculated using the dry film thickness.

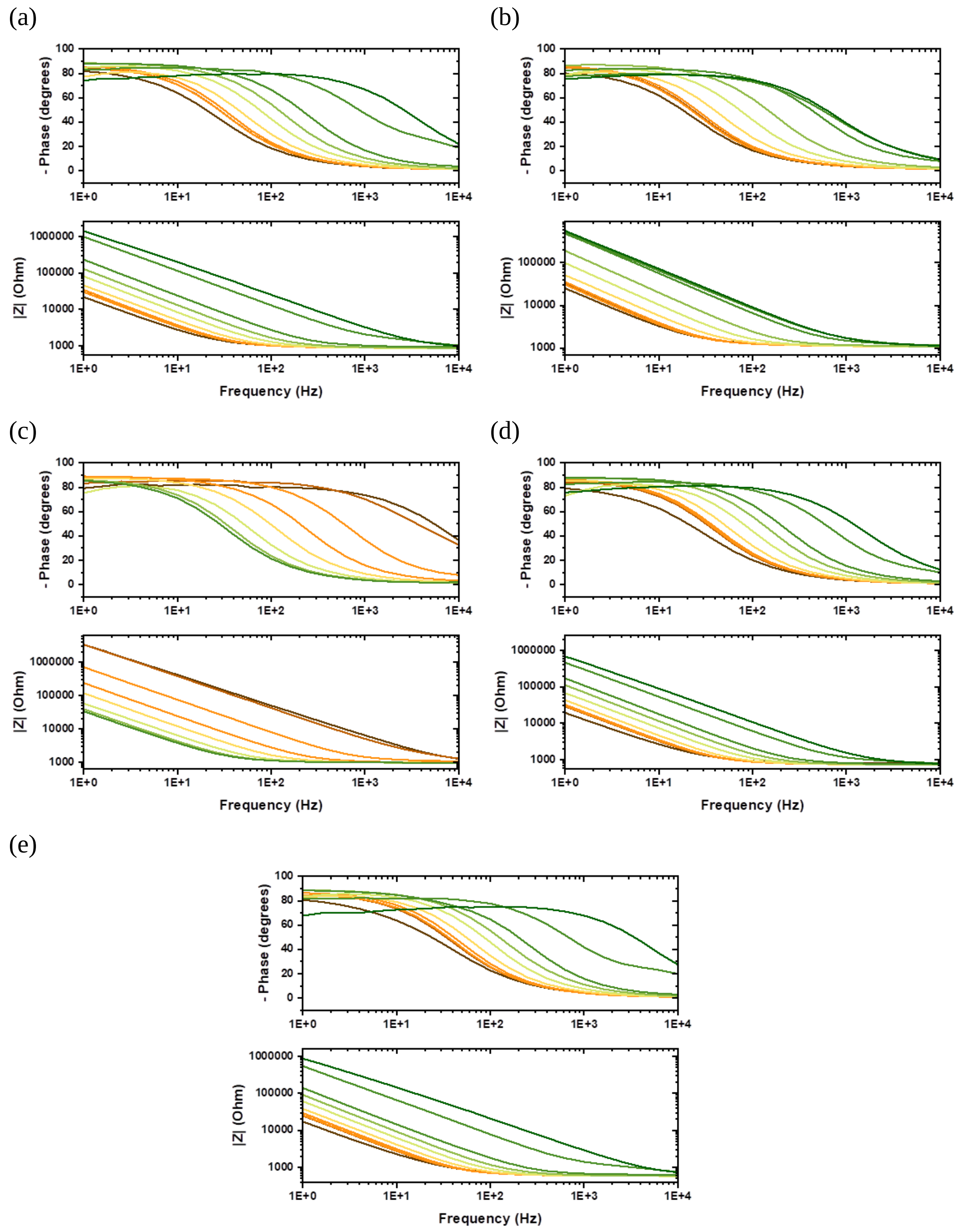

Supplementary Figure 33. Electrochemical impedance spectroscopy of (a) unpurified and (b)-(e) fractions 1-4 of p(C_6g_3NDI-T) showing the impedance magnitude and the phase of the impedance at V_G = 0.8 to 0 V. *C** was calculated using the dry film thickness.

Stability measurements

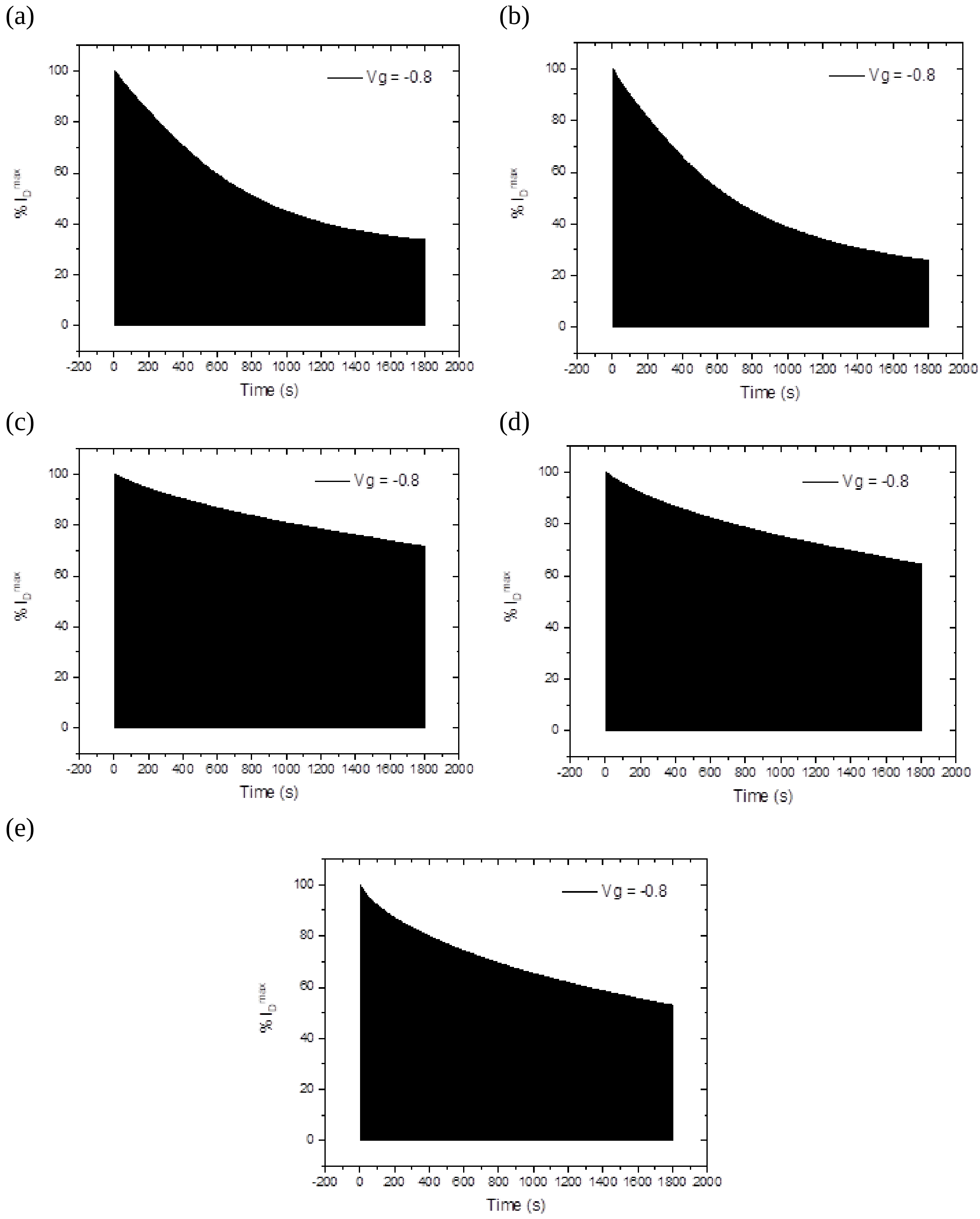

Supplementary Figure 34. Operational stability of p(g_4T2-TT) (a) unpurified and (b)-(e) fractions 1-4, respectively, over 30 minutes.

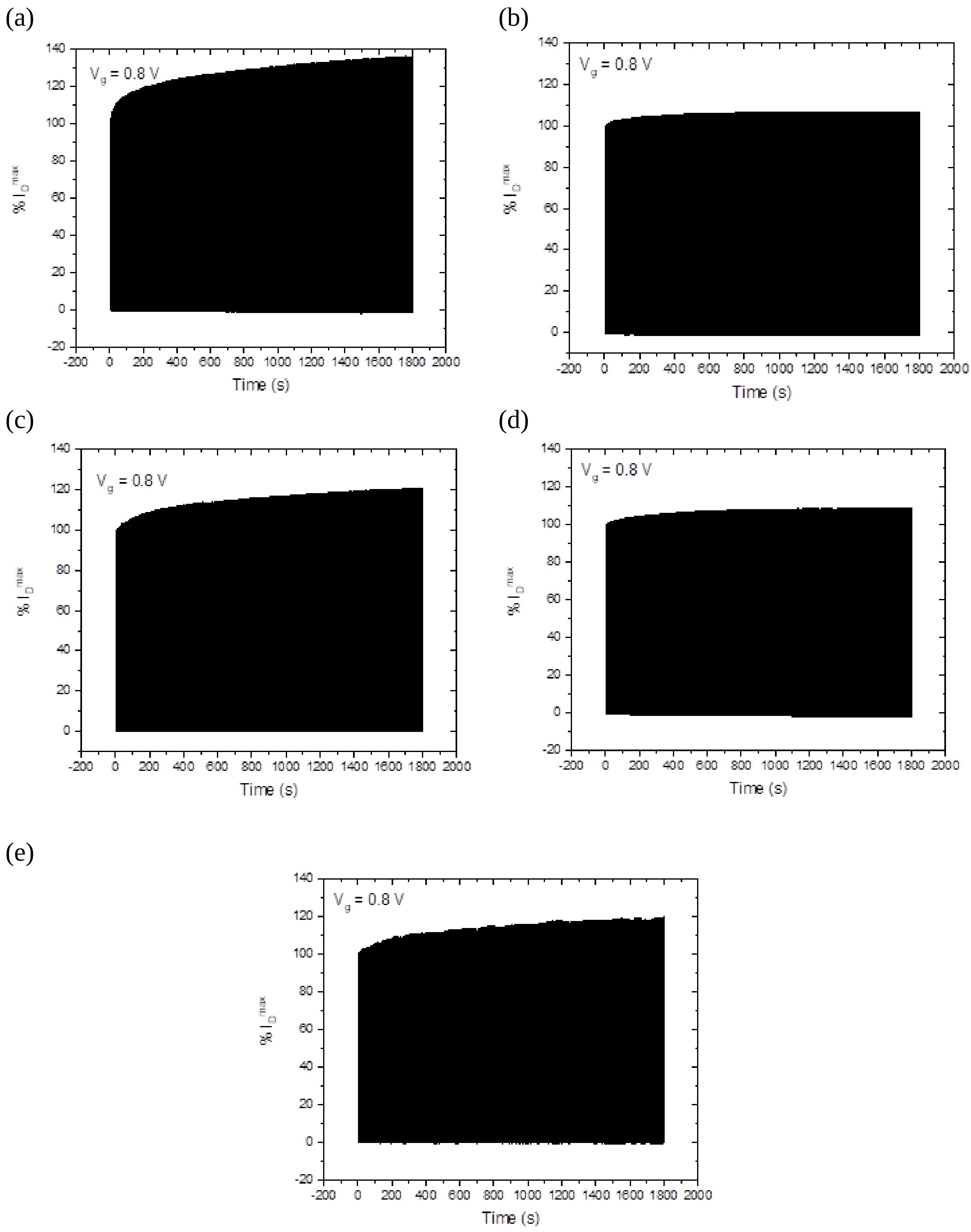

Supplementary Figure 35. Operational stability of p(C_6g_3NDI-T) (a) unpurified and (b)-(e) fractions 1-4, respectively, over 30 minutes.

SUPPLEMENTARY TABLE

Table S1. Threshold voltages for each fraction extracted from a plot of $\sqrt{I_D}$ versus V_G in OECT devices.

**Polymer**		V_{TH} [V]
**p(g$_4$T2-TT)**	Unpurifed	0.16 ± 0.014
	F2	0.041 ± 0.015
	F1	0.085 ± 0.008
	F3	0.067 ± 0.003
	F4	0.019 ± 0.002
	F3 + $Pd_2(dba)_3$	0.10 ± 0.010
	Recombined	-0.076 ± 0.007
**p(C$_6$g$_3$NDI-T)**	Unpurifed	0.29 ± 0.005
	F4	0.24 ± 0.003
	F1	0.27 ± 0.014
	F2	0.22 ± 0.020
	F2 + $Pd_2(dba)_3$	0.36 ± 0.005
	Recombined	0.28 ± 0.004

SUPPLEMENTARY DISCUSSION

Methods for and justification of measuring molecular weight. The molecular weight can be measured in various ways, namely through absolute or relative determination. The most common methods include size exclusion chromatography (SEC), which includes gel permeation chromatography (GPC),[1–3] matrix-assisted laser desorption/ionization-time of flight (MALDI-ToF)[4,5] and nuclear magnetic resonance (NMR) end group analysis.[6] Each method comes with limitations, for example, GPC is typically a relative technique, calibrated to polystyrene standards, which may not be a good comparison for polymers with different hydrodynamic volumes. The resulting inaccuracies make it difficult to compare different polymers by this method. Furthermore, data may be inflated in the case of aggregation, or poor data analysis can lead to over-integration of bimodal distributions. MALDI-ToF likely underestimates M_n as heavier polymer chains may not 'fly'. Similarly, inaccuracies rise for high molecular weight polymers characterized by NMR end group analysis as deterministic end groups may not be detectable. On top of this, NMR end group analysis often involves significant extra synthetic workload to develop model systems for comparison.

SUPPLEMENTARY METHODS

Monomer synthesis. All reactions were performed in oven-dried glassware under a nitrogen atmosphere using standard Schlenk techniques.

Monomer synthesis of g_4T2-Br_2

3,3'-bis((2,5,8,11-tetraoxatridecan-13-yl)oxy)-2,2'-bithiophene (g_4T2)

Copper(I) iodide (0.470 g, 2.47 mmol) and potassium tert-butoxide (4.16 g, 37.10 mmol) were dissolved in tetraethylene glycol (24 mL, 123.32 mmol) under a nitrogen atmosphere and stirred at room temperature for 30 minutes. To this, 3,3'-dibromo-2,2'-bithiophene (4.00 g, 12.34 mmol) and the reaction mixture was heated to 100 °C overnight. The reaction mixture was cooled to room temperature and washed through a frit with DCM (200 mL). The filtrate was then washed with a solution of ammonium chloride (2x 150 mL), a solution of 1 M HCl (2x 150 mL), water (2x 150mL) then brine (150 mL). The organic phase was dried over magnesium sulfate and excess solvent was removed *in vacuo*. The crude product was purified by silica column chromatography employing a 1:1 v/v mixture of ethyl acetate:petroleum ether as the eluent (R_f = 0.25). The final product was obtained as a pale yellow oil (4.54 g, 7.84 mmol, 64% yield): ^{1}H NMR (400 MHz, Chloroform-*d*) δ 7.21 (dd, *J* = 5.5, 1.8 Hz, 2H), 6.97 (dd, *J* = 5.5, 1.9 Hz, 1H), 6.90 (dd, *J* = 5.7, 2.0 Hz, 1H), 4.32 (t, *J* = 5.6Hz, 2H), 3.84 (dd, *J* = 5.6, 4.3 Hz, 2H), 3.76 – 3.58 (m, 12H), 3.59 – 3.48 (m, 2H), 3.35 (s, 3H); ^{13}C NMR (101 MHz, Chloroform-*d*) δ 154.3, 130.6, 130.1, 124.8, 123.8, 116.9, 113.2, 107.6, 72.0, 71.5, 71.0, 70.7, 70.7, 70.6, 70.0, 59.1. HRMS (ES-ToF): 579.2217 [$M+H^+$] (calc. 579.2220).

13,13'-((5,5'-dibromo-[2,2'-bithiophene]-3,3'-diyl)bis(oxy))bis(2,5,8,11-tetraoxatridecane) (g_4T2Br_2)

g_4T2 (4.49 g, 7.76 mmol) was dissolved in anhydrous THF (120 mL) under a nitrogen atmosphere. The reaction mixture was cooled to 0 °C and kept in the dark, while N-bromosuccinimide (3.04 g, 17.08 mmol) was added in four portions over 10 minutes. The solution was stirred at room temperature for 40 minutes, before the addition of DCM (150 mL). The reaction mixture was quenched with an aqueous solution of sodium metabisulfite and sodium hydrogen carbonate (3x 80 mL), then washed with water (3x 100 mL) and dried over sodium sulfate. Excess solvent was removed under reduced pressure. The crude material was purified by silica column chromatography employing a 1:1 v/v mixture of ethyl acetate:petroleum ether as the solvent system (R_f = 0.40). The final product was obtained as a colourless oil (5.16 g, 6.75 mmol, 87% yield): ^{1}H NMR (400 MHz, Acetone-d_6) δ 7.15 (s, 2H), 4.33 – 4.26 (m, 3H), 3.90 – 3.82 (m, 5H), 3.67 (ddd, *J* = 5.9, 3.9, 1.1 Hz, 5H), 3.66 – 3.52 (m, 18H), 3.50 – 3.41 (m, 2H), 3.27 (s, 6H); ^{13}C NMR (101 MHz, Acetone-d_6) δ 206.1, 121.0, 72.7, 72.6, 71.4, 71.4, 71.3, 71.1, 70.6, 58.8. HRMS (ES-ToF): 735.0437 [$M+H^+$] (calc. 735.0430).

Monomer synthesis of 4,9-dibromo-2,7-di(2,5,8,11-tetraoxaheptadecan-17-yl)benzo[lmn][3,8]phenanthroline-1,3,6,8(2H,7H)-tetraone (C_6g_3NDI-Br_2)

*Hexane-1,6-diyl bis(4-methylbenzenesulfonate) (**1**)*

Hexane-1,6-diol (10.0 g, 84.6 mmol, 1.0 equiv.) was dissolved in pyridine (40 mL) under ambient conditions. The reaction mixture was cooled to 0 °C and 4-toluenesulfonyl chloride (35.5 g, 186 mmol, 3.1 equiv.) was added in 4 portions, then stirred at room temperature for 30

minutes. The mixture was stored in the fridge overnight, then poured into 250 mL 2M HCl. The mixture was extracted with DCM (3 x 250 mL) and the organic fraction was washed with water (3 x 200 mL) then brine (1 x 200 mL) and dried over anhydrous Na_2SO_4. The solvent was removed *in vacuo* and the crude product was recrystallised from methanol to obtain the product as a white crystalline solid (31.14 g, 73.01 mmol, 86% yield): ^{1}H NMR (400 MHz, $CDCl_3$) δ 7.77 (d, *J* = 8 Hz, 4H), 7.34 (d, *J* = 8 Hz, 4H), 3.98 (t, *J* = 6.4 Hz, 4H), 2.45 (s, 6H), 1.63 – 1.55 (m, 4H), 1.30 – 1.23 (m, 4H); ^{13}C NMR (101 MHz, $CDCl_3$) δ 144.9, 133.3, 130.0, 128.0, 70.4, 28.8, 24.9, 21.8. HRMS (ESI-ToF): 427.5410 [M+H$^+$] (calc. 427.5418).

*2,5,8,11-tetraoxaheptadecan-17-yl 4-methylbenzenesulfonate (**2**)*

Sodium hydride (60%, 2.38 g, 59.5 mmol, 1.5 equiv.) was dissolved in dry THF (50 mL) and the reaction mixture was cooled to 0 °C. Triethylene glycol monomethyl ether (6.35 mL, 39.7 mmol, 1.0 equiv.) was added dropwise, then the mixture was brought to room temperature and stirred for 30 minutes. The reaction mixture was heated to 65 °C for 1 hour, then cooled back to 0 °C. Separately, hexane-1,6-diyl bis(4-methylbenzenesulfonate) (16.9 g, 39.7 mmol, 1.0 equiv.) was dissolved in dry THF (50 mL) and this mixture was transferred to the first solution via canula. The reaction mixture was then stirred at room temperature for 1 hour, then heated to 55 °C overnight. The reaction was then cool to room temperature and poured over ice water. This mixture was extracted with chloroform (2 x 150 mL), then the organic portion was washed with water (3 x 150 mL) and brine (1 x 150 mL) and dried over Na_2SO_4. The solvent was removed *in vacuo* and the crude product was purified by silica column chromatography employing a 2:1 v/v mixture of ethyl acetate:petroleum ether as the solvent system (R_f = 0.30). The final product was dried by high vacuum and obtained as a colourless oil (5.65 g, 13.5 mmol, 34% yield): ^{1}H NMR (400 MHz, $CDCl_3$) δ 7.76 – 7.69 (m, 2H), 7.33 – 7.26 (m, 2H), 3.96 (t, *J* = 6.5 Hz, 2H), 3.63 – 3.55 (m, 8H), 3.54 – 3.47 (m, 4H), 3.36 (t, *J* = 6 Hz, 2H), 3.32 (s, 3H), 2.40 (s, 3H), 1.64 – 1.41 (m, 4H), 1.34 – 1.16 (m, 4H); ^{13}C NMR (101 MHz, $CDCl_3$) δ 144.6, 133.2, 129.8, 127.8, 71.9, 71.1, 70.6, 70.5, 70.4, 70.1, 59.0, 29.4, 28.7, 25.5, 25.2, 21.6. HRMS (ESI-ToF): 419.5460 (calc. 419.5402).

*17-azido-2,5,8,11-tetraoxaheptadecane (**3**)*

2,5,8,11-tetraoxaheptadecan-17-yl 4-methylbenzenesulfonate (3.75 g, 8.96 mmol, 1.0 equiv.) was dissolved in dry DMF (35 mL) and cool to 0 °C. To this, sodium azide (0.870 g, 13.4 mmol, 1.5 equiv.) was added and the mixture was heated to 100 °C for 2 hours. Subsequently, the reaction mixture was cooled to room temperature and ethyl acetate (200 mL) was added. The precipitate was filtered and the filtrate was washed with water (3 x 200 mL) and brine (1 x 150 mL) and dried over Na_2SO_4, before the solvent was removed *in vacuo*. The crude product was pure and used directly without further purification as a colourless oil (2.23 g, 7.71 mmol, 86% yield): ^{1}H NMR (400 MHz, $CDCl_3$) 3.59 – 3.49 (m, 8H), 3.49 – 3.39 (m, 4H), 3.33 (t, *J* = 6.6 Hz, 2H), 3.25 (s, 3H), 3.14 (t, *J* = 6.9 Hz, 2H), 1.48 (ddt, *J* = 8.2, 5.4, 1.2 Hz, 4H), 1.33 – 1.20 (m, 4H); ^{13}C NMR (101 MHz, $CDCl_3$) δ 71.8, 71.0, 70.4, 70.4, 70.3, 69.9, 58.8, 51.2, 29.3, 28.6, 26.4, 25.5. HRMS (ESI-ToF): 290.3760 [M+H$^+$] (calc. 290.3761).

2,5,8,11-tetraoxaheptadecan-17-amine (4)

17-azido-2,5,8,11-tetraoxaheptadecane (2.20 g, 7.60 mmol, 1.0 equiv.) was dissolved in dry THF (40 mL) and cooled to 0 °C. Triphenylphosphine (2.99g, 11.4 mmol,1.5 equiv.) was added

in and the mixture was stirred at room temperature for 2 hours. The reaction mixture was heated to 50 °C for 1 hour, then returned to room temperature, before deionised water (0.2 mL) was added. The reaction was stirred overnight at room temperature, then diluted with water (200 mL). The aqueous portion was washed with diethyl ether (3 x 200 mL), then concentrated *in vacuo* to obtain the pure product as a pale yellow oil (1.82 g, 69.2 mmol, 91% yield): ^{1}H NMR (400 MHz, $CDCl_3$) δ 3.71 – 3.59 (m, 9H), 3.59 – 3.50 (m, 4H), 3.43 (t, J = 6.7 Hz, 2H), 3.36 (s, 3H), 2.67 (t, J = 7.0 Hz, 2H), 1.56 (m, 2H), 1.47 – 1.39 (m, 2H), 1.38 – 1.28 (m, 4H); ^{13}C NMR (101 MHz, $CDCl_3$) δ 71.9, 71.4, 70.6, 70.6, 70.5, 70.1, 59.0, 42.1, 33.5, 29.5, 26.7, 26.0. HRMS (ESI-ToF): 264.3769 [M+H$^+$] (calc. 264.3780).

4,9-dibromo-2,7-di(2,5,8,11-tetraoxaheptadecan-17-yl)benzo[lmn][3,8]phenanthroline-1,3,6,8(2H,7H)-tetraone (C_6g_3NDI-Br_2)

The procedure for preparing the monomer was modified as follows. 2,6-dibromonaphthalene-1,4,5,8-tetracarboxylic dianhydride (NDABr_2, 1.60 g, 3.76 mmol) and zinc acetate (1.04 g, 7.51 mmol) were dissolved in anhydrous *o*-xylene (80 mL). The reaction mixture was heated to 90 °C, then 2,5,8,11-tetraoxaheptadecan-17-amine (2.08 g, 7.89 mmol) was added dropwise. The solution was heated to 125 °C for 2 hours, then cooled to room temperature and diluted with chloroform (200 mL). The reaction mixture was washed with water (3x 150 mL), dried over magnesium sulfate and the excess solvent was removed under reduced pressure. The crude product was purified using silica column chromatography with ethyl acetate as the solvent system (R_f = 0.25). The final product was obtained as a pale yellow solid (0.683 g, 0.745 mmol, 20% yield): ^{1}H NMR (400 MHz, Chloroform-*d*) δ 8.99 (s, 2H), 4.22 – 4.16 (m, 4H), 3.68 – 3.62 (m, 16H), 3.60 – 3.52 (m, 8H), 3.46 (t, J = 6.6 Hz, 4H), 3.37 (s, 6H), 1.75 (m, 4H), 1.61 (t, J = 6.8 Hz, 4H), 1.44 (d, J = 3.7 Hz, 8H); ^{13}C NMR (101 MHz, Chloroform-*d*) δ 160.9, 139.2, 127.9, 125.5, 72.1, 71.4, 70.8, 70.8, 70.7, 70.2, 59.2, 41.7, 29.7, 28.0, 27.1, 26.0. HRMS (ES-ToF): 915.2196 [M+H$^+$] (calc. 915.2200).

Polymer synthesis

Polymer synthesis of p(g_4T2-TT)

g_4T2-Br_2 (525.74 mg, 0.714 mmol), 2,5-bis(trimethylstannyl)-thieno[3,2-*b*]thiophene (332.52 mg, 0.714 mmol) and tetrakis(triphenylphosphine)palladium(0) (16.50 mg, 14.278 µmol) were dissolved in 1:1 v/v mixture of anhydrous degassed dimethylformamide:chlorobenzene (9.6 mL total volume). The resulting solution was degassed for an additional 10 minutes and the reaction was heated 110 °C overnight. The crude polymer solution was cooled to room temperature and precipitated into methanol, and the polymer was purified utilising Soxhlet extractions with: hexane, ethyl acetate, acetone, methanol, tetrahydrofuran and dichloromethane. The polymer was then dissolved in chloroform and excess solvent was removed *in vacuo*. The final product was collected by reprecipitating the polymer into methanol and collecting it by suction filtration. p(g_4T2-TT) was obtained as a dark blue solid (393.05 mg, 0.550 mmol, 77% yield): ^{1}H NMR (400 MHz, DMSO-d_6) δ 7.33 (br s), 7.09 (br s), 4.37 (br s), 3.91-3.44 (br m), 3.27 (br s); GPC (DMF, 40 °C): M_n = 83.4 kDa, M_w = 112.7 kDa.

Polymer synthesis of p(C_6g_3NDI-T)

C_6g_3NDI-Br_2 (306.50 mg, 0.334 mmol), 2,5-bis(trimethylstannyl)thiophene (137.00 mg, 0.334 mmol), tris(dibenzylideneacetone)dipalladium(0) (6.12 mg, 6.683 µmol) and tris(*o*-methoxyphenyl)phosphine (9.45 mg, 26.74 µmol) were dissolved in anhydrous degassed chlorobenzene (6 mL). The resulting solution was degassed for an additional 10 minutes and the reaction was heated 130 °C overnight. The crude polymer solution was cooled to room temperature and precipitated into a 2:1 v/v mixture of ethyl acetate:hexane, and the polymer was purified via Soxhlet extractions with: hexane, ethyl acetate, acetone, methanol, tetrahydrofuran and dichloromethane. The polymer was then dissolved in chloroform and excess solvent was removed *in vacuo*. The final product was collected by reprecipitating the polymer into a 2:1 v/v mixture of ethyl acetate:hexane and collecting it by suction filtration. p(C_6g_3NDI-T) was obtained as a dark blue solid (264.43 mg, 0.304 mmol, 91% yield): ^{1}H NMR (400 MHz, Chloroform-*d*) δ 8.97 (s), 7.47 (br s), 4.19 (br s), 3.65 – 3.62 (m), 3.58-3.52 (m), 3.45 (t), 3.36 (s), 1.63-1.59 (br m), 1.44 (br s); GPC (chloroform, 40 °C): M_n = 42.4 kDa, M_w = 87.4 kDa.

Polymer fractionation. The polymer was fractionated by preparative gel permeation chromatography in chloroform, at a concentration of 5 mg/mL. The polymer was recycled for one cycle and collected into four fractions on the second cycle, where each fraction corresponds to approximately a quarter of the material. The preparative GPC traces for the fractionation can be found in Figure 1.

SUPPLEMENTARY REFERENCES

1. Moser, M. *et al.* Propylene and butylene glycol: new alternatives to ethylene glycol in conjugated polymers for bioelectronic applications. *Mater. Horizons* **9**, 973–980 (2022).

2. Feng, K. *et al.* Fused Bithiophene Imide Dimer-Based n-Type Polymers for High-Performance Organic Electrochemical Transistors. *Angew. Chemie Int. Ed.* **60**, 24198–24205 (2021).

3. Savagian, L. R. *et al.* Balancing Charge Storage and Mobility in an Oligo(Ether) Functionalized Dioxythiophene Copolymer for Organic- and Aqueous- Based Electrochemical Devices and Transistors. *Adv. Mater.* **30**, 1804647 (2018).

4. Giovannitti, A. *et al.* The Role of the Side Chain on the Performance of N-type Conjugated Polymers in Aqueous Electrolytes. *Chem. Mater.* **30**, 2945–2953 (2018).

5. Ohayon, D. *et al.* Influence of Side Chains on the n-Type Organic Electrochemical Transistor Performance. *ACS Appl. Mater. Interfaces* **13**, 4253–4266 (2021).

6. Shin, Y. H. *et al.* Synthesis and Aggregation Behavior of a Glycolated Naphthalene Diimide Bithiophene Copolymer for Application in Low-Level n-Doped Organic Thermoelectrics. *Macromolecules* **53**, 5158–5168 (2020).
